# Comparative Effectiveness of Enhanced Patient Instructions for Bowel Preparation Before Colonoscopy: Network Meta-analysis of 23 Randomized Controlled Trials

**DOI:** 10.2196/19915

**Published:** 2021-10-25

**Authors:** Xu Tian, Li-Juan Yi, Yang Han, Hui Chen, Xiao-Ling Liu, Wei-Qing Chen, Maria F Jiménez-Herrera

**Affiliations:** 1 Nursing Department Universitat Rovira i Virgili Tarragona Spain; 2 Department of Nursing Hunan Traditional Chinese Medical College Zhuzhou China; 3 Chongqing University Cancer Hospital Chongqing China

**Keywords:** colonoscopy, bowel preparation, patient instruction, systematic review, network meta-analysis

## Abstract

**Background:**

Various enhanced patient instructions (EPIs) have been used for bowel preparation (BP) and our previous meta-analysis also demonstrated the efficacy of EPIs in increasing the colonic polyp and adenoma detection rates; however, the optimal method for adequate BP has not yet been developed.

**Objective:**

We performed a network meta-analysis to determine the optimal instructions.

**Methods:**

We searched for randomized controlled trials (RCTs) comparing the effectiveness of EPIs with each other or standard patient instructions (SPIs) for BP. We performed direct and Bayesian network meta-analyses for all instructions and used the GRADE (Grading of Recommendations Assessment, Development and Evaluation) criteria to appraise the quality of evidence.

**Results:**

We included 23 RCTs (7969 patients) comparing 10 different instructions. In direct meta-analyses, most of the EPIs, except visual aids and mobile apps, increased the adequate preparation rate (APR). Network meta-analyses showed that additional explanations were superior to visual aids (odds ratio [OR] 0.35, 95% CI 0.19-0.59), telephone calls (OR 0.62, 95% CI 0.37-0.99), educational videos (OR 0.79, 95% CI 0.5-0.77), and mobile apps (OR 0.33, 95% CI 0.14-0.68) with low-to-high-quality evidence; newly designed booklets (OR 3.28, 95% CI 1.59-6.16), SMS text messaging (OR 2.33, 95% CI 1.28-3.91), telephone calls (OR 1.86, 95% CI 1.03-1.78), educational videos (OR 2.33, 95% CI 1.40-3.65), and social media applications (OR 2.42, 95% CI 1.4-3.93) were superior to visual aids and mobile apps with low-to-high-quality evidence. SMS text messaging, telephone calls, and social media applications increase adherence to and satisfaction with the BP regime. Social media applications reduce the risk of adverse events (AEs). Telephone calls and social media applications increase the polyp detection rate (PDR).

**Conclusions:**

Newly designed booklets, telephone calls, educational videos, and social media applications can improve the quality of BP. Telephone calls and social media applications improve adherence to and satisfaction with the BP regime, reduce the risk of AEs, and increase the PDR.

**Trial Registration:**

INPLASY (International Platform of Registered Systematic Review and Meta-analysis Protocols) INPLASY2020120103; https://inplasy.com/inplasy-2020-12-0103/

## Introduction

Colorectal cancer ranks third among the most common cancers and second in terms of the incidence and cause of cancer-related mortality worldwide, with 1.8 million new cases and 0.88 million deaths in 2018 [1]. Colonoscopy has been regarded as the criterion standard approach for early detection and safe removal of colorectal lesions [2-4]. In particular, screening colonoscopy has been associated with decreased colorectal cancer incidence and mortality [2,5]. The quality of bowel preparation (BP) is an important contributor toward successful and safe colonoscopy [6]. However, approximately 18% to 30.5% inadequate BP has been reported in previous studies [7-9]. It is discouraging that inadequate BP is associated with decreased polyp detection rates (PDRs), increased risk of adverse events (AEs), prolonged working time, and increased medical expenditure [10,11].

Previous studies have determined several factors that can influence the quality of BP, such as appropriate dietary restrictions and proper administration of preparation solutions [12]. Of all the factors, adequate comprehension of the BP and colonoscopy details is a critical contributor to adequate BP [13]. Patients usually receive written booklets or verbal instructions from professionals before colonoscopy for details regarding BP and dietary restrictions, which are defined as standard patient instructions (SPIs) [14]. However, the effect of SPIs on improving the quality of BP is not enough [15]. Therefore, researchers and practitioners have been developing most of the enhanced patient instructions (EPIs) by including cartoon pictures, SMS text messaging, telephone calls, mobile apps, and social media applications to improve the quality of BP prior to colonoscopy [14].

Thus far, several traditional pairwise meta-analyses investigating the comparative efficacy between EPIs and SPIs for the quality of BP have been published, and they have demonstrated improved BP [13,14,16-18]. Moreover, our previous meta-analysis also demonstrated the efficacy of EPIs in increasing the PDR and adenomas detection rate (ADR) [19]. However, only 2 3-arm randomized controlled trials (RCTs) investigated the comparative efficacy of telephone calls or WeChat versus SMS text messaging for BP in patients receiving outpatient colonoscopy. It is still unclear which EPIs should preferably be used by decision makers for BP before colonoscopy. To address those issues that could not be addressed by traditional pairwise meta-analysis, network meta-analysis, which can simultaneously assess the comparative efficacy of multiple interventions, has been developed [20,21]. Therefore, we performed direct pairwise and Bayesian network meta-analyses combining direct and indirect evidence comparing the relative efficacy of all patient instructions to determine the optimal educational instructions for BP before colonoscopy. We also used the GRADE (Grading of Recommendations Assessment, Development and Evaluation) criteria to appraise the quality of evidence.

## Methods

We conducted this systematic review and network meta-analysis in accordance with the PRISMA (Preferred Reporting Items for Systematic Reviews and Meta-Analyses) statement [22] and reported all the outcomes according to the International Society for Pharmacoeconomics and Outcomes Research Task Force on Indirect Treatment Comparisons Good Research Practices [23]. No formal protocol for the present study has been published. We registered our systematic review on the INSPLAY (International Platform of Registered Systematic Review and Meta-analysis Protocols) platform, and the trial registration number is INPLASY2020120103.

### Search Strategy

We constructed the search strategy with the assistance of an experienced medical librarian using full-text words and MeSH (Medical Subject Headings). We also refined the search strategy according to the specific requirements of each database. All potential RCTs comparing EPIs to each other or SPIs for BP were captured in PubMed, the Cochrane Central Register of Controlled Trials, and Embase until December 2019. The last search was updated in February 2020. Details of the search strategies used for the 3 targeted databases are presented in Multimedia Appendix 1.

### Study Selection

We first excluded duplicate records through running the Finding Duplicate function embedded in EndNote (version X9, Clarivate Analytics). Then, we checked the titles and abstracts of the articles to exclude irrelevant articles. Next, we screened the full texts to further check the eligibility of all the remaining studies. The inclusion criteria were as follows: (1) patients: adult patients who were assigned to receive selective outpatient colonoscopy; (2) interventions: all EPIs or SPIs for BP; (3) outcomes: the quality of BP assessed with the adequate preparation rate (APR), adherence to instruction (AI), satisfaction with the BP solution, willingness to repeat the same BP solution, PDR, and AEs including abdominal discomfort, nausea and vomiting, and sleep disturbance; (4) study design: RCTs. Language restrictions were not imposed. The exclusion criteria included (1) animal studies and (2) conference abstracts without sufficient data or unpublished studies.

The eligibility was checked by 2 investigators (XT and HC) independently, and any divergences were resolved through the consensus principle. When no agreement could be reached, a third investigator (WQC) was consulted for determining the eligibility.

### Data Extraction

Essential data including the leading author, year of publication, study design, country where the study was conducted, age and sex of the patients, details of the BP regime, details of the instructions used, and outcomes of interest were extracted by 2 independent investigators (LJY and XT) using a data extraction sheet designed in advance. EPIs were classified as additional explanations, visual aids, new visual aids, newly designed booklets, SMS text messages, telephone calls, mobile apps, social media applications, and educational videos. The classification and comparison of EPIs are documented in Multimedia Appendix 2.

The primary outcome of the present meta-analysis was the comparative efficacy of EPIs for improving the quality of BP before colonoscopy, which was assessed with respect to the APR. The secondary outcome was the comparative efficacy of EPIs with respect to the AI, satisfaction with the BP solution, willingness to repeat the same BP solution, PDR, and AEs.

### Quality Assessment

We assigned 2 investigators to independently assess the risk of bias of each eligible study with the Cochrane risk of bias assessment tool [24]. We labeled each study as having low, unclear, or high risk of bias according to the match between the actual information and the following assessment criteria: random sequence generation, allocation concealment, blinding of participants and personnel, blinding of outcome assessment, incomplete outcome data, selective reporting, and other biases. A third investigator (WQC) was consulted to solve any discrepancies.

### Statistical Analysis

In traditional pairwise meta-analysis, we calculated the pooled odds ratio (OR) with 95% CI to express the dichotomous data [25]. We performed Cochran Q tests to qualitatively assess the heterogeneity and used the *I*^2^ statistic to quantitatively estimate the level of heterogeneity [26]. All pairwise meta-analyses were performed based on the random-effect model because this model simultaneously incorporates within- and between-study heterogeneities. Publication bias is assessed using a funnel plot if the accumulated number of eligible studies for individual outcomes was more than 10 [27], and an asymmetry suggests the presence of publication bias [28]. Direct meta-analysis was conducted using Review Manager 5.3 (Cochrane Collaboration).

After completing direct meta-analysis, we conducted random-effect network meta-analyses to estimate all the relative effects using Markov chain Monte Carlo methods in OpenBUGS 3.2.3 (MRC Biostatistics Unit) following the methods described by Lu and Ades [29,30]. We used the initial value that was automatically generated from the software to fit the model [31]. To achieve convergence, we performed each Markov chain Monte Carlo method with 50,000 iterations and 20,000 burn-ins. We drew the comparison-adjusted funnel plot to assess the small-study effects when the number of studies included in one comparison pair exceeded 10 [32].

We assessed the probability that each instruction was the most efficacious one for improving quality of BP, the second best, the third best, and so on by calculating the OR for each instruction compared with an arbitrary common control group and counting the number of iterations of the Markov chain in which each instruction had the highest OR, the second highest OR, and so on [33].

### Sensitivity Analysis

We designed several sensitivity analyses to evaluate the robustness of the summarized findings according to the following principles: (1) BP assessment scale (excluding studies in which uncommon scales were used except for the Boston Bowel Preparation Scale [BBPS], Ottawa Bowel Preparation Scale [OBPS], and Aronchick Bowel Preparation Scale [ABPS]); (2) risk of bias (excluding studies with high risk); (3) study design (excluding studies with multicenter design).

### Quality of Evidence

We rated the quality of evidence of the primary outcomes with the GRADE working group approach [34,35]. In this approach, the quality of direct evidence based on RCTs would be first rated as high and the level could be reduced to moderate, low, or very low according to 5 domains, including risk of bias, indirectness, imprecision, inconsistency, and publication bias [35]. The quality of indirect evidence was consistent with the level of the lowest rating of the 2 pairwise estimates that contribute as first-order loops to the indirect estimates and imprecision or intransitivity can further reduce the level [35]. If the assumption of coherence between direct and indirect estimates was confirmed, then the higher one of their levels would be assigned to the results from network meta-analysis [35].

## Results

### Study Selection

**Figure 1:**
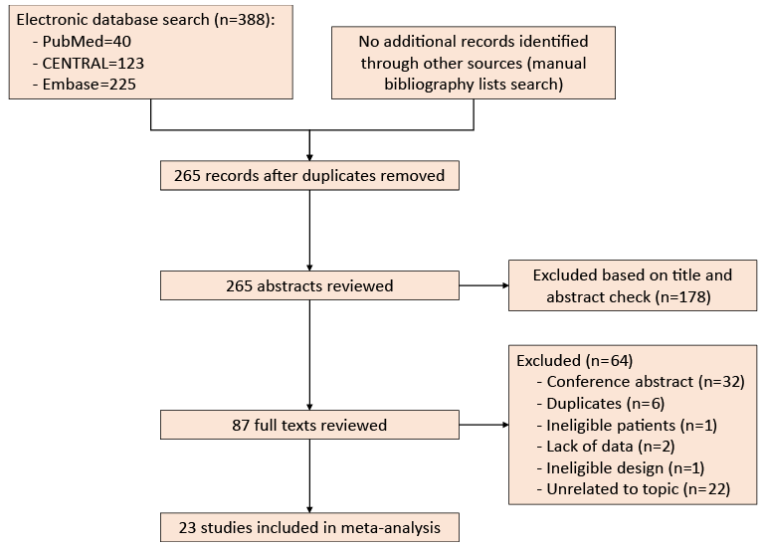
Flow diagram showing retrieval and selection of literature. CENTRAL: Cochrane Central Register of Controlled Trials.

Figure 1 presents the schematic flowchart of the study selection process. From a total of 388 unique studies identified using the search strategy, we included 23 RCTs in this network meta-analysis [36-58]. Among these, 1 RCT involving a newly designed educational booklet was excluded (because of inpatient enrollment) [59]. Further, 1 RCT comparing modified BP protocols (multimedia education) to standard BP protocols was excluded owing to the design implementation using historical control data [60]. Moreover, 2 RCTs comparing educational videos or additional explanations with SPIs were excluded owing to the lack of essential data [61,62].

### Study Characteristics

The basic characteristics of the patients who were included in all eligible studies are summarized in Table 1. We have also documented the characteristics of the eligible studies in Multimedia Appendix 3. The studies were published between 2009 and 2019 [36-56,58]. The sample sizes of the individual studies ranged from 92 to 1018 with a total of 7969 participants. Among the 23 studies included, 2 were designed as 3-arm trials [41,55]; among these, Lee et al [41] compared the efficacy between SMS text messaging, telephone calls, and SPIs, and Wang et al compared the efficacy between SMS text messaging, social media applications and SPIs [55]. All other studies had 2-arm designs [36-40,42-54,56,58]. Further, 2 studies had multicenter designs [49,54]. All the studies reported the quality of BP assessed using the BBPS (9 trials) [36,41,45,50,53-57], OBPS (8 trials) [39,40,42,43,47-49,52], ABPS (4 trials) [37,38,51,58], Harefield Cleansing Scale (1 trial) [44], and Universal Preparation Assessment Scale (1 trial) [46].

**Table 1:** Basic characteristics of the patients included in studies on educational instructions for bowel preparation before colonoscopy.

Study	Country	Comparison	Sample size (male vs female participants)	Age (years, male vs female participants), mean (SD)	Male vs female participants (%)	Outcomes
Back et al 2018 [36]	Korea	Educational videos vs SPIs^a^ (verbal instructions and instructional leaflets)	283 (139 vs 144)	55.4 (12.8) vs 57.6 (13.1)	53.4 vs 56.2	APR^b^, AI^c^, and SE^d^
Calderwood et al 2011 [57]	United States	Visual aids vs SPIs (written information)	969 (477 vs 492)	57.3 (8.0) vs 57.1 (7.3)	41.5 vs 41.7	APR, CIT^e^, WT^f^, PDR^g^, and AEs^h^
Cho et al 2015 [58]	Korea	Educational videos vs SPIs (verbal education)	101 (51 vs 50)	n.r.^i^	52.9 vs 52	APR
Elvas et al 2017 [37]	Portugal	Additional explanations vs SPIs (oral and written information)	229 (116 vs 113)	60.0 (13.0) vs 59.0 (11.0)	50.9 vs 58.4	APR and WRBP^j^
Garg et al 2016 [38]	United States	Educational videos vs SPIs (verbal education)	94 (48 vs 46)	59.3 (18.1) vs 57.3 (19.4)	43.8 vs 45.7	APR, PDR, ADR^k^, CIT, and WT
Jeon et al 2018 [36]	Korea	Educational videos vs SPIs (written information)	281 (140 vs 141)	46.7 (9.9) vs 49.9 (9.6)	57.1 vs 57.4	APR, AI, PDR, ADR, CIT, WT AEs, and SDT^l^
Kang et al 2016 [40]	China	Social media applications vs SPIs (verbal and written instructions)	770 (387 vs 383)	45.5 (13.0) vs 44.4 (13.2)	52.2 vs 49.9	APR, AI, WRBP, ADR, CIT, WT, ICIBP^m^, AEs, and SDT
Lee et al 2015 [41]	South Korea	SMS text messaging vs telephone calls vs SPIs (written information)	390 (127 vs 126 vs 137)	45.7 (12.4) vs 46.0 (12.2) vs 47.1 (11.8)	59.8 vs 62.7 vs 53.3	APR, CIR^n^, AI, SE, WRBP, PDR, ADR, CIT, WT, ICIBP, AEs, and SDT
Liu et al 2018 [42]	China	Educational videos vs SPIs (written information)	281 (239 vs 237)	55.1 (6.3) vs 54.4 (8.6)	61.5 vs 64.9	APR, PDR, CIT, WT, and ICIBP
Liu et al 2014 [43]	China	Telephone calls vs SPIs (verbal and written instructions)	605 (300 vs 305)	44.8 (12.5) vs 45.7 (12.6)	53.3 vs 48.2	APR, AI, WRBP, PDR, CIR, CIT, WT, AEs, and SDT
Lorenzo et al 2015 [44]	Spain	Mobile app vs SPIs (written information)	260 (108 vs 152)	48.3 (13.5) vs 52.5 (14.0)	44.4 vs 40.1	APR, AI, SE, and WRBP
Meng 2015 [45]	China	Additional explanations vs SPIs (verbal education)	618 (318 vs 300)	59.0 (15.7)	n.r.	APR and AI
Modi et al 2009 [46]	United States	Additional explanations vs SPIs (written and verbal instructions)	164 (84 vs 80)	57.9 (9.1) vs 57.3 (9.1)	45.2 vs 33.8	APR, CIT, and WT
Park et al 2016 [47]	South Korea	Educational videos vs SPIs (oral and written information)	502 (250 vs 252)	49.2 (8.6) vs 47.3 (9.2)	62.8 vs 66.3	APR, PDR, CIT, and WT
Pillai et al 2018 [48]	United States	Educational videos vs SPIs (verbal and written instructions)	104 (56 vs 48)	n.r.	44.6 vs 50	APR
Prakash et al 2013 [49]	United States	Educational videos vs SPIs (written information)	133 (67 vs 66)	n.r.	52.2 vs 36.4	APR and SE
Sharara et al 2017 [51]	Lebanon	Mobile app vs SPIs (written information)	160 (80 vs 80)	52.0 (13.0) vs 55.0 (12.0)	60 vs 46.3	APR and AI
Spiegel et al 2011 [52]	United States	Newly designed booklet vs SPIs (written information)	436 (216 vs 220)	60.0 (10.7) vs 60.0 (12.3)	95.8 vs 97.7	APR
Tae et al 2012 [53]	Korea	New visual aids vs SPIs (oral and written information)	205 (102 vs 103)	48.6 (8.8) vs 47.6 (9.2)	71.6 vs 68.9	APR, PDR, CIT, CST, and WT
Walter et al 2019 [54]	Germany	SMS text messaging vs SPIs (oral and written information)	495 (248 vs 247)	47.5 (13.6) vs 47.2 (14.8)	50.8 vs 46.9	APR, PDR, ADR, CST, and WT
Wang et al 2019 [55]	China	SMS text messaging vs social media applications vs SPIs (oral and written information)	384 (129 vs 128 vs 127)	52.6 (12.7) vs 48.9 (13.0) vs 51.5 (12.1)	54.3 vs 61.7 vs 53.5	APR, AI, SE, WRBP, PDR, ADR, CIR, CIT, WT, AEs, and SDT
Zhang et al 2018 [56]	China	Social media applications vs SPIs (oral and written information)	1018 (511 vs 507)	51.2 (18.5) vs 50.7 (17.9)	52.6 vs 50.1	APR, AI, WRBP, ADR, CIR, CIT, WT, AEs, and SDT
Rice et al 2016 [50]	United States	Educational videos vs SPIs (oral and written information)	92 (42 vs 50)	60.1 (8.8) vs 61.0 (7.9)	61.9 vs 50.4	APR

^a^SPIs: standard patient instructions.

^b^APR: adequate preparation rate.

^c^AI: adherence to instruction.

^d^SE: satisfaction with the education.

^e^CIT: cecal intubation time.

^f^WT: withdrawal time.

^g^PDR: polyp detection rate.

^h^AEs: adverse events.

^i^n.r.: not reported.

^j^WRBP: willingness to repeat the same BP solution.

^k^ADR: adenomas detection rate.

^l^SDT: sleep disturbance.

^m^ICIBP: incomplete colonoscopy due to inadequate bowel preparation.

^n^CIR: cecal intubation rate.

### Quality Assessment

The overall and study-level quality assessments are outlined in Figure S1 of Multimedia Appendix 4. Overall, most of the studies (18/23, 78.3%) appeared to have been at low-to-moderate risk of bias, with 4 (17.4%) not reporting the details of generating the random sequence [45,47,49,56] and 8 (34.8%) not reporting the details for allocation concealment [36,38,45-48,53,57]. Further, 10 (43.5%) did not report the details of blinding the outcome assessor [39-41,43-45,47,51,53,54]. More importantly, 4 studies (17.4%) did not blind the assessors and were rated to be at high risk of bias [38,46,48,49]. In addition, 4 (17.4%) were rated to be at high risk of attrition bias [38,46,49,53]. Other bias sources were not detected in all studies.

### Direct Treatment Effects

#### Primary Outcomes

**Figure 2:**
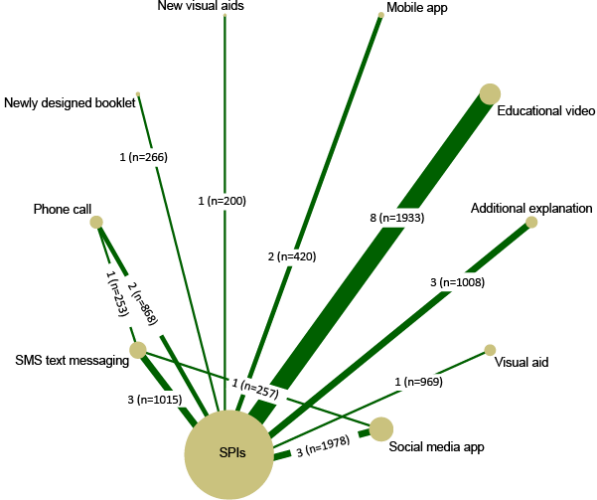
Evidence network for adequate preparation rate. The numbers outside and inside parentheses refer to the number of trials and the total number of participants in these trials, respectively, and the thickness of the connecting lines corresponds to the number of trials between comparators. SPIs: standard patient instructions.

Figure 2 demonstrates the available direct comparisons and network meta-analyses. Compared to SPIs, additional explanations (3 RCTs; OR 3.34, 95% CI 1.45-7.69), newly designed booklets (1 RCT; OR 3.63, 95% CI 2.15-6.12), new visual aids (1 RCT; OR 3.05, 95% CI 1.21-7.68), SMS text messaging (3 RCTs; OR 2.77, 95% CI 1.86-4.14), telephone calls (2 RCTs; OR 2.64, 95% CI 1.03-6.74), educational videos (8 RCTs; OR 2.82, 95% CI 1.83-4.35), and social media applications (3 RCTs; OR 2.70, 95% CI 1.75-4.18) adequately increased the BP rate, but not visual aids (1 RCT; OR 1.18, 95% CI 0.78-1.8) and mobile apps (2 RCTs; OR 1.85, 95% CI 0.15-23.24). Head-to-head meta-analysis showed that telephone calls (1 RCT; OR 1.25, 95% CI 0.33-4.77) or social media applications (1 RCT; OR 1.32, 95% CI 0.63-2.78) were not superior to SMS messaging. Significant heterogeneity was observed in trials comparing additional explanations (*I^2^*=79%), telephone calls (*I^2^*=60%), educational videos (*I^2^*=62%), and mobile apps (*I^2^*=68%). All the pooled results are delineated in Figure S2 in Multimedia Appendix 4.

#### Secondary Outcomes

Compared to SPIs, additional explanations (1 RCT; OR 8.38, 95% CI 3.73-18.84), SMS text messaging (2 RCTs; OR 7.04, 95% CI 2.79-17.81), telephone calls (2 RCTs; OR 4.54, 95% CI 2.92-7.05), and social media applications (3 RCTs; OR 3.88, 95% CI 2.05-7.35) are associated with improved AI, but not educational videos (1 RCT; OR 3.16, 95% CI 0.99-10.06) or mobile apps (2 RCTs; OR 1.26, 95% CI 0.73-2.18). Head-to head meta-analysis showed that telephone calls (1 RCT; OR 1.25, 95% CI 0.33-4.77) and social media applications (1 RCT; OR 0.86, 95% CI 0.3-2.45) were not superior to SMS text messaging. Significant heterogeneity was observed in trials comparing social media applications (*I^2^*=63%) to SPIs. All the pooled results are delineated in Figure S3 in Multimedia Appendix 4.

Compared to SPIs, social media applications (1 RCT; OR 2.56, 95% CI 1.35-4.87) and telephone calls (1 RCT; OR 1.85, 95% CI 1.01-3.41) indicated improved satisfaction with the instructions but not SMS text messaging (2 RCTs; OR 1.92, 95% CI 0.94-4.05) or educational videos (1 RCT; OR 1.47, 95% CI 0.69-3.12). Head-to-head meta-analysis showed that telephone calls (1 RCT; OR 1.22, 95% CI 0.73-2.06) and social media applications (1 RCT; OR 0.95, 95% CI 0.52-1.72) were not superior to SMS text messaging. All the pooled results are delineated in Figure S4 in Multimedia Appendix 4.

Compared to SPIs, social media applications (3 RCTs; OR 2.16, 95% CI 1.51-3.09) and mobile apps (1 RCT; OR 2.48; 95% CI 1.22-5.04) were associated with improved willingness to repeat the same BP regime, but not additional explanations (1 RCT; OR 2.04, 95% CI 0.37-11.35), SMS text messaging (2 RCTs; OR 1.24, 95% CI 0.57-2.71), or telephone calls (2 RCTs; OR 1.58, 95% CI 0.67-3.69). Head-to-head meta-analysis showed that telephone calls (1 RCT; OR 1.44, 95% CI 0.61-3.37) and social media applications (1 RCT; OR 0.99, 95% CI 0.44-2.23) were not superior to SMS text messaging. Significant heterogeneity was observed in trials comparing telephone calls (*I^2^*=69%) to SPIs. All the pooled results are delineated in Figure S5 in Multimedia Appendix 4.

Compared to SPIs, telephone calls (2 RCTs; OR 1.85, 95% CI 1.36-2.5) were associated with an increased PDR, but not visual aids (1 RCT; OR 0.99, 95% CI 0.76 -1.28), new visual aids (1 RCT; OR 0.99, 95% CI 0.57-1.73), SMS text messaging (3 RCTs; OR 1.07, 95% CI 0.81-1.41), educational videos (5 RCTs; OR 1.05, 95% CI 0.72-1.54), or social media applications (1 RCT; OR 1.25, 95% CI 0.65-2.41). Head-to-head meta-analysis showed that telephone calls (1 RCT; OR 1.61, 95% CI 0.97-2.68) were not superior to SMS text messaging. Significant heterogeneity was observed in the trials comparing educational videos (*I^2^*=52%) to SPIs. All the pooled results are delineated in Figure S6 in Multimedia Appendix 4.

Compared to SPIs, social media applications were associated with reduced risk of abdominal discomfort (3 RCTs; OR 0.67, 95% CI 0.5-0.9), but not visual aids (1 RCT; OR 0.9, 95% CI 0.63-1.28), SMS text messaging (2 RCTs; OR 0.67, 95% CI 0.4-1.12), telephone calls (2 RCTs; OR 0.94, 95% CI 0.65-1.35), or educational videos (1 RCT; OR 1.01, 95% CI 0.37-2.76). Head-to-head meta-analysis showed that telephone calls (1 RCT; OR 0.83, 95% CI 0.47-1.48) or social media applications (1 RCT; OR 1.22, 95% CI 0.61-2.45) were not superior to SMS text messaging. All the pooled results are delineated in Figure S7 in Multimedia Appendix 4.

Compared to SPIs, social media applications indicated reduced risk of nausea and vomiting (3 RCTs; OR 0.7, 95% CI 0.55-0.88), but not visual aids (1 RCT; OR 1.27, 95% CI 0.67-2.41), SMS text messaging (2 RCTs; OR 0.64, 95% CI 0.27-1.53), telephone calls (2 RCTs; OR 0.8, 95% CI 0.56-1.13), or educational videos (1 RCT; OR 0.49, 95% CI 0.14-1.66). Head-to-head meta-analysis showed that telephone calls (1 RCT; OR 0.55, 95% CI 0.33-0.91) were superior to SMS text messaging, but not social media applications (1 RCT; OR 1.19, 95% CI 0.7-2.03). Significant heterogeneity was observed in the trials comparing SMS text messaging (*I^2^*=74%) to SPIs. All the pooled results are delineated in Figure S8 in Multimedia Appendix 4.

Compared to SPIs, SMS text messaging (2 RCTs; OR 1.1, 95% CI 0.71-1.71), telephone calls (2 RCTs; OR 0.69, 95% CI 0.41-1.16), educational videos (1 RCT; OR 2.32, 95% CI 0.78-6.86), and social media applications (3 RCTs; OR 0.62, 95% CI 0.35-1.10) were not associated with increased risk of sleep disturbance. Head-to-head meta-analysis showed that telephone calls (1 RCT; OR 1.08, 95% CI 0.65-1.8) and social media applications (1 RCT; OR 1.08, 95% CI 0.66-1.77) were not superior to SMS text messaging. Significant heterogeneity was observed in the trials comparing telephone calls (*I^2^*=55%) and social media applications (*I^2^*=85%) to SPIs. All the pooled results are delineated in Figure S9 in Multimedia Appendix 4.

### Network Meta-Analysis and Quality Assessment

#### Primary Outcomes

For the primary outcome of APR, the results of direct and indirect comparisons were largely similar with overlapping CIs, although differences were observed in the effect size and statistical significance in some cases, as shown in Table 2. In the network meta-analysis, we calculated the mixed-effect estimate as the weighted average of the direct (where available) and indirect treatment effects. In this analysis, when compared with SPIs, additional explanations (OR 3.56,95% CI 2.46-5; moderate quality of evidence), newly designed booklets (OR 3.81, 95% CI 2.19-6.3; very low quality of evidence), new visual aids (OR 3.61, 95% CI 1.29-8.63; very low quality of evidence), SMS text messaging (OR 2.7, 95% CI 1.85-3.86; high quality of evidence), telephone calls (OR 2.15, 95% CI 1.49-3.02; moderate quality of evidence), educational videos (OR 2.7, 95% CI 2.12-3.41; low quality of evidence), and social media applications (OR 2.81, 95% CI 2.07-3.73; high quality of evidence) increased the APR in patients undergoing colonoscopy, but not visual aids (OR 1.21, 95% CI 0.78-1.81; moderate quality of evidence) and mobile apps (OR 1.15, 95% CI 0.53-2.19; very low quality of evidence), as observed in Table 2.

Network meta-analysis demonstrated that visual aids (OR 0.35, 95% CI 0.19-0.59; high quality of evidence), telephone calls (OR 0.62, 95% CI 0.37-0.99; high quality of evidence), educational videos (OR 0.79, 95% CI 0.5-0.77; moderate quality of evidence), and mobile apps (OR 0.33, 95% CI 0.14-0.68; low quality of evidence) were inferior to additional explanations; newly designed booklets (OR 3.28, 95% CI 1.59-6.16; low quality of evidence), SMS text messaging (OR 2.33, 95% CI 1.28-3.91; high quality of evidence), telephone calls (OR 1.86, 95% CI 1.03-1.78; high quality of evidence), educational videos (OR 2.33, 95% CI 1.4-3.65; moderate quality of evidence), and social media applications (OR 2.42, 95% CI 1.4-3.93; high quality of evidence) were superior to visual aids; mobile apps were inferior to newly designed booklets (OR 0.32, 95% CI 0.12-0.7; low quality of evidence) or SMS text messaging (OR 0.44, 95% CI 0.18-0.9; low quality of evidence); social media applications (OR 0.43, 95% CI 0.19-0.85; low quality of evidence) were inferior to educational videos, and mobile apps (OR 0.42, 95% CI 0.18-0.83; low quality of evidence) were inferior to social media applications in increasing the APR.

**Table 2:** Pooled summary estimates and quality of evidence derived from direct and indirect estimates and network meta-analysis informing on comparative efficacy of educational interventions for improving quality of bowel preparation before colonoscopy^a^.

Educational instruction		Direct estimate				Indirect estimate				Network meta-analysis		
		OR^b^ (95% CI)		Quality of evidence		OR (95% CI)		Quality of evidence		OR (95% CI)		Quality of evidence
**Compared with standard patient instructions**												
	Additional explanations	*3.34 (1.45-7.69)*	Moderate^c^		n.e.^d^		N/A^e^		*3.56 (2.46-5)*		Moderate	
	Visual aids	*1.18 (0.78-1.8)*	Moderate^f^		n.e.		N/A		1.21 (0.78-1.81)		Moderate	
	Newly designed booklets	*3.63 (2.15-6.12)*	Very low^g,h^		n.e.		N/A		*3.81 (2.19-6.3)*		Very low	
	New visual aids	*3.05 (1.21-7.68)*	Very low^c,g,i^		n.e.		N/A		*3.61 (1.29-8.63)*		Very low	
	SMS text messaging	*2.77 (1.86-4.14)*	High		2.12 (0.96-4.03)		Very low		*2.7 (1.85-3.86)*		High	
	Telephone calls	*2.64 (1.03-6.74)*	Moderate^j^		2.22 (0.55-8.93)		Very low		*2.15 (1.49-3.02)*		Moderate	
	Educational videos	*2.82 (1.83-4.35)*	Low^k^		n.e.		N/A		*2.7 (2.12-3.41)*		Low	
	Social media applications	*2.7 (1.75-4.18)*	High		1.03 (0.57-1.85)		Low		*2.81 (2.07-3.73)*		High	
	Mobile apps	1.85 (0.15-23.24)	Very low^j,h^		n.e.		N/A		1.15 (0.53-2.19)		Very low	
**Compared with additional explanations**												
	Visual aids	N/A	N/A		*0.34 (0.14-0.87)*		Moderate		*0.35 (0.19-0.59)*		High	
	Newly designed booklets	N/A	N/A		1.05 (0.39-2.83)		Very low		1.11 (0.56-2)		Very low	
	New visual aids	N/A	N/A		0.89 (0.26-3.08)		Very low		1.05 (0.34-2.61)		Very low	
	SMS text messaging	N/A	N/A		0.81 (0.32-2.03)		Moderate		0.79 (0.45-1.26)		Moderate	
	Telephone calls	N/A	N/A		0.77 (0.22-2.7)		Moderate		*0.62 (0.37-0.99)*		High	
	Educational videos	N/A	N/A		0.82 (0.32-2.1)		Low		*0.79 (0.5-0.77)*		Moderate	
	Social media applications	N/A	N/A		0.79 (0.31-2.01)		Moderate		0.81 (0.5-1.26)		Moderate	
	Mobile apps	N/A	N/A		0.54 (0.04-7.66)		Very low		*0.33 (0.14-0.68)*		Low	
**Compared with visual aids**												
	Newly designed booklets	N/A	N/A		*3.08 (1.58-6.01)*		Very low		*3.28 (1.59-6.16)*		Low	
	New visual aids	N/A	N/A		2.59 (0.94-7.13)		Very low		3.12 (0.98-7.96)		Very low	
	SMS text messaging	N/A	N/A		*2.35 (1.32-4.19)*		Moderate		*2.33 (1.28-3.91)*		High	
	Telephone calls	N/A	N/A		2.24 (0.8-6.26)		Moderate		*1.86 (1.03-1.78)*		High	
	Educational videos	N/A	N/A		*2.39 (1.31-4.36)*		Low		*2.33 (1.4-3.65)*		Moderate	
	Social media applications	N/A	N/A		*2.29 (1.25-4.18)*		Moderate		*2.42 (1.4-3.93)*		High	
	Mobile apps	N/A	N/A		1.57 (0.86-2.87)		Very low		0.99 (0.4-2.05)		Very low	
**Compared with newly designed booklets**												
	New visual aids	N/A	N/A		0.84 (0.29-2.43)		Very low		1.02 (0.3-2.68)		Very low	
	SMS text messaging	N/A	N/A		0.76 (0.4-1.47)		Very low		0.76 (0.38-1.37)		Very low	
	Telephone calls	N/A	N/A		0.73 (0.25-2.13)		Very low		0.61 (0.3-1.09)		Very low	
	Educational videos	N/A	N/A		0.78 (0.39-1.53)		Very low		0.76 (0.41-1.3)		Very low	
	Social media applications	N/A	N/A		0.74 (0.38-1.47)		Very low		0.79 (0.41-1.37)		Very low	
	Mobile apps	N/A	N/A		0.51 (0.04-6.69)		Very low		*0.32 (0.12-0.7)*		Low	
**Compared with new visual aids**												
	SMS text messaging	N/A	N/A		0.91 (0.33-2.49)		Very low		0.94 (0.29-2.24)		Very low	
	Telephone calls	N/A	N/A		0.87 (0.23-3.23)		Very low		0.75 (0.23-1.76)		Very low	
	Educational videos	N/A	N/A		0.93 (0.33-2.57)		Very low		0.95 (0.3-2.15)		Very low	
	Social media applications	N/A	N/A		0.89 (0.32-2.46)		Very low		0.98 (0.31-2.27)		Very low	
	Mobile apps	N/A	N/A		0.61 (0.04-8.9)		Very low		0.4 (0.1-1.05)		Very low	
**Compared with SMS text messaging**												
	Telephone calls	1.25 (0.33-4.77)	Very low^f,i^		0.95 (0.34-2.65)		Moderate		0.82 (0.48-1.31)		Moderate	
	Educational videos	N/A	N/A		1.02 (0.57-1.84)		Low		1.04 (0.65-1.56)		Low	
	Social media applications	1.32 (0.63-2.78)	Low^l^		0.98 (0.54-1.76)		High		1.07 (0.67-1.62)		High	
	Mobile apps	N/A	N/A		0.67 (0.05-8.58)		Very low		*0.44 (0.18-0.9)*		Low	
**Compared with telephone calls**												
	Educational videos	N/A	N/A		1.07 (0.38-3.01)		Low		1.3 (0.83-1.95)		Low	
	Social media applications	N/A	N/A		1.02 (0.36-2.88)		Moderate		1.35 (0.82-2.07)		Moderate	
	Mobile apps	N/A	N/A		0.70 (0.05-10.33)		Very low		0.55 (0.23-1.13)		Very low	
**Compared with educational videos**												
	Social media applications	N/A	N/A		0.96 (0.52-1.77)		Low		1.05 (0.71-1.51)		Low	
	Mobile apps	N/A	N/A		0.66 (0.05-8.47)		Very low		*0.43 (0.19-0.85)*		Low	
**Compared with social media applications**												
	Mobile apps	N/A	N/A		0.69 (0.05-8.85)		Very low		*0.42 (0.18-0.83)*		Low	

^a^The italicized values indicate statistically significant differences.

^b^OR: odds ratio.

^c^One was rated with high risk.

^d^n.e.: not estimable.

^e^N/A: not applicable.

^f^Only one was captured.

^g^Point estimates between two studies were conflicting.

^h^One with only 266 participants was included.

^i^One with only 200 participants was included.

^j^A wide 95% CI was generated.

^k^Two were rated with high risk.

^l^One with only 257 participants was included.

For the primary outcome of APR, newly designed booklets had the highest probability of being ranked the best (85.8%), followed by additional explanations (80.6%), new visual aids (71.6%), social media applications (64.6%), educational videos (61.0%), SMS text messaging (60.4%), telephone calls (40.9%), visual aids (15.9), mobile apps (12%), and SPIs (7.2%), as shown in Figure S10 in Multimedia Appendix 4.

#### Secondary Outcomes

Network meta-analysis showed that when compared to SPIs, additional explanations (OR 9.84, 95% CI 4.13-21.78), SMS text messaging (OR 6.99, 95% CI 3.57-12.92), telephone calls (OR 8.48, 95% CI 3.08-7.42), educational videos (OR 4.24, 95% CI 1.12-12.61), and social media applications (OR 3.76, 95% CI 2.7-5.13) increased adherence to the preparation regime, but not mobile apps (OR 1.33, 95% CI 0.74-2.2), as shown in Table 3. Additional explanations significantly increased adherence to the preparation regime when compared to social media applications (OR 0.46, 95% CI 0.16-0.96) or mobile apps (OR 0.16, 95% CI 0.05-0.37). Moreover, SMS messaging (OR 0.21, 95% CI 0.08-0.44), telephone calls (OR 0.29, 95% CI 0.13-0.54), and social media applications (OR 0.36, 95% CI 0.18-0.65) significantly increased adherence to the preparation regime compared to mobile apps.

**Table 3:** Pooled relative risks of secondary outcomes based on combined direct and indirect evidence from Bayesian network meta-analysis with different educational instructions in patients undergoing colonoscopy^a^.

Education instruction		Adherence to instruction	Satisfaction with BP_b_	Willingness to repeat	Abdominal discomfort	Nausea and vomiting	Sleep disturbance	Polyp detection rate
**Compared with standard patient instructions**								
	Additional explanations	*9.84 (4.13-21.78)*	N/A^c^	1.02 (0.19-3.24)	N/A	N/A	N/A	N/A
	Visual aids	N/A	N/A	N/A	0.91 (0.62-1.27)	1.35 (0.67-2.46)	N/A	1 (0.76-1.28)
	New visual aids	N/A	N/A	N/A	N/A	N/A	N/A	1.03 (0.56-1.74)
	SMS text messaging	*6.99 (3.57-12.92)*	*1.79 (1.22-2.55)*	1.44 (0.86-2.32)	0.71 (0.46-1.03)	*0.66 (0.46-0.9)*	0.8 (0.58-1.1)	1.1 (0.85-1.41)
	Telephone calls	*4.84 (3.08-7.42)*	*1.97 (1.19-3.12)*	1.36 (0.95-1.89)	0.95 (0.67-1.31)	0.92 (0.67-1.25)	*0.69 (0.51-0.91)*	*1.86 (1.4-2.44)*
	Educational videos	*4.24 (1.12-12.61)*	1.6 (0.7-3.18)	N/A	1.18 (0.36-2.93)	2.77 (0.65-8.51)	2.88 (0.84-7.93)	0.81 (0.6-1.07)
	Social media applications	*3.76 (2.7-5.13)*	*2.03 (1.17-3.34)*	*2.22 (1.77-2.75)*	*0.69 (0.52-0.9)*	*0.69 (0.54-0.85)*	*0.53 (0.44-0.65)*	1.37 (0.87-2.01)
	Mobile apps	1.33 (0.74-2.2)	N/A	*2.73 (1.27-5.34)*	N/A	N/A	N/A	N/A
**Compared with additional explanations**								
	SMS text messaging	0.85 (0.25-2.07)	N/A	2.39 (0.4-8.14)	N/A	N/A	N/A	N/A
	Telephone calls	0.59 (0.2-1.29)	N/A	2.25 (0.4-7.44)	N/A	N/A	N/A	N/A
	Educational videos	0.52 (0.09-1.74)	N/A	N/A	N/A	N/A	N/A	N/A
	Social media applications	*0.46 (0.16-0.96)*	N/A	3.68 (0.66-12.1)	N/A	N/A	N/A	N/A
	Mobile apps	*0.16 (0.05-0.37)*	N/A	4.53 (0.67-16.48)	N/A	N/A	N/A	N/A
**Compared with visual aids**								
	New visual aids	N/A	N/A	N/A	N/A	N/A	N/A	1.05 (0.54-1.87)
	SMS text messaging	N/A	N/A	N/A	0.8 (0.46-1.32)	0.54 (0.24-1.05)	N/A	1.13 (0.77-1.59)
	Telephone calls	N/A	N/A	N/A	1.08 (0.64-1.7)	0.76 (0.35-1.45)	N/A	*1.90 (1.28-2.72)*
	Educational videos	N/A	N/A	N/A	1.34 (0.37-3.47)	2.28 (0.43-7.59)	N/A	0.82 (0.55-1.19)
	Social media applications	N/A	N/A	N/A	0.79 (0.49-1.2)	0.57 (0.27-1.05)	N/A	1.39 (0.82-2.2)
**Compared with new visual aids**								
	SMS text messaging	N/A	N/A	N/A	N/A	N/A	N/A	1.16 (0.6-2.04)
	Telephone calls	N/A	N/A	N/A	N/A	N/A	N/A	1.96 (1-3.46)
	Educational videos	N/A	N/A	N/A	N/A	N/A	N/A	0.85 (0.43-1.52)
	Social media applications	N/A	N/A	N/A	N/A	N/A	N/A	1.44 (0.67-2.71)
**Compared with SMS text messaging**								
	Telephone calls	0.77 (0.33-1.49)	1.12 (0.66-1.79)	0.99 (0.55-1.65)	1.39 (0.86-2.14)	1.44 (0.95-2.1)	0.88 (0.59-1.27)	*1.71 (1.2-2.36)*
	Educational videos	0.67 (0.14-2.19)	0.93 (0.36-1.95)	N/A	1.74 (0.48-4.55)	4.34 (0.95-13.54)	*3.68 (1.01-10.24)*	0.74 (0.49-1.07)
	Social media applications	0.59 (0.28-1.09)	1.16 (0.65-1.92)	1.64 (0.93-2.65)	1.02 (0.63-1.57)	1.07 (0.73-1.52)	*0.68 (0.47-0.95)*	1.25 (0.79-1.88)
	Mobile apps	*0.21 (0.08-0.44)*	N/A	2.02 (0.77-4.42)	N/A	N/A	N/A	N/A
**Compared with telephone calls**								
	Educational videos	0.92 (0.22-2.87)	0.86 (0.32-1.89)	N/A	1.28 (0.37-3.32)	3.07 (0.69-9.65)	*4.24 (1.17-11.87)*	*0.44 (0.29-0.65)*
	Social media applications	0.82 (0.45-1.34)	1.09 (0.52-2.01)	*1.69 (1.1-2.48)*	0.75 (0.48-1.12)	0.76 (0.51-1.09)	0.79 (0.55-1.1)	0.75 (0.44-1.17)
	Mobile apps	*0.29 (0.13-0.54)*	N/A	2.08 (0.88-4.29)	N/A	N/A	N/A	N/A
**Compared with educational videos**								
	Social media applications	1.31 (0.28-3.5)	1.48 (0.53-3.3)	N/A	0.78 (0.48-1.97)	0.38 (0.08-1.08)	*0.26 (0.07-0.64)*	*1.73 (1.01-2.77)*
	Mobile apps	0.46 (0.09-1.32)	N/A	N/A	N/A	N/A	N/A	N/A
**Compared with social media applications**								
	Mobile apps	*0.36 (0.18-0.65)*	N/A	1.25 (0.56-2.5)	N/A	N/A	N/A	N/A

^a^The italicized values indicate statistically significant differences.

^b^BP: bowel preparation.

^c^N/A: not applicable.

The analysis also demonstrated that besides educational videos, SMS text messaging (OR 1.79, 95% CI 1.22-2.55), telephone calls (OR 1.97, 95% CI 1.19-3.12), and social media applications (OR 2.03, 95% CI 1.17-3.34) indicated satisfaction with the BP regime when compared to SPIs.

Network meta-analysis also showed that when compared to SPIs, social media applications (OR 2.22, 95% CI 1.77-2.75) or mobile apps (OR 2.73, 95% CI 1.27-5.34) were associated with increased willingness to repeat the same BP regime. Social media applications (OR 1.69, 95% CI 1.1-2.48) significantly increased the willingness to repeat the same BP regime when compared to telephone calls.

Furthermore, telephone calls significantly increased the PDR when compared to SPIs (OR 1.86, 95% CI 1.4-2.44) and SMS messaging (OR 1.71, 95% CI 1.2-2.36). Social media applications (OR 1.73, 95% CI 1.01-2.77) were associated with an increased PDR compared to educational videos.

The analysis also showed that besides social media applications (OR 0.69, 95% CI 0.54-0.85), no other instructions were associated with decreased abdominal discomfort, when compared to SPIs. SMS text messaging (OR 0.66, 95% CI 0.46-0.9) or social media applications (OR 0.69, 95% CI 0.54-0.85) were superior to SPIs in reducing the risk of nausea and vomiting. Telephone calls or social media applications were associated with reduced risk of sleep disturbance but not SMS text messaging or educational videos, when compared to SPIs. Moreover, SMS text messaging (OR 3.68, 95% CI 1.01-10.24) or telephone calls (OR 4.24, 95% CI 1.17-11.87) were also superior to educational videos in improving sleep disturbance. Social media applications were also superior to SMS text messaging (OR 0.68, 95% CI 0.47-0.95) or educational videos (OR 0.26, 95% CI 0.07-0.64) in reducing the risk of sleep disturbance

### Sensitivity Analysis

Results from multiple prespecified sensitivity analyses are presented in Multimedia Appendix 5. Overall, for the primary outcome, the results were largely similar to the main analysis in the sensitivity analyses based on the (1) BP assessment scale (excluding studies in which uncommon scales were used except for BBPS, OBPS, and ABPS), (2) risk of bias (excluding studies with high risk), and (3) study design (excluding studies with multicenter designs). After excluding the studies using uncommon BP assessment scales, new visual aids (OR 3.16, 95% CI 1.02-8.01) were statistically superior to visual aids or mobile apps (OR 0.27, 95% CI 0.06-0.75) in increasing the APR; the difference (OR 0.49, 95% CI 0.2-1.03) between mobile apps and social media applications was not significant in increasing adherence to the BP regime. Excluding the studies with high risk also showed that telephone calls (OR 0.63, 95% CI 0.37-1.01) and educational videos (OR 0.79, 95% CI 0.5-1.19) were not inferior to additional explanations in increasing the APR. Excluding studies using uncommon preparation assessment scales showed that social media applications were not superior to mobile apps (OR 0.49, 95% CI 0.2-1.03) in increasing adherence to the BP regime. Furthermore, excluding the studies with high risk revealed that the difference (OR 1.59, 95% CI 0.88-2.66) between mobile apps and social media applications in increasing the PDR was not significant.

### Publication Bias and Network Coherence

**Figure 3:**
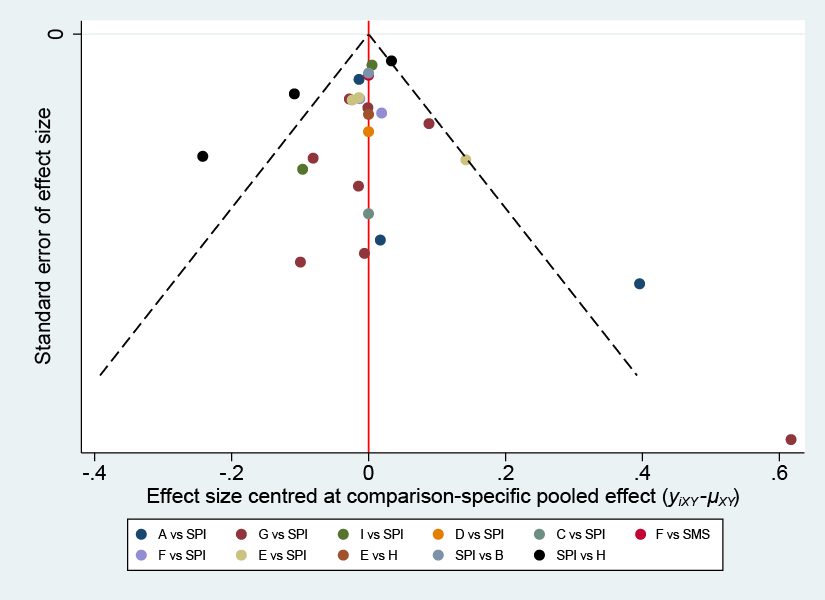
Comparison-adjusted funnel plot for adequate preparation rate. The vertical axis represents the standard error of the effect size. The horizontal axis indicates the effect size centered at the comparison-specific pooled effect. The symmetrical funnel plot indicates the absence of publication bias. PDR: polyp detection rate; EPI: enhanced patient instruction; SPI: standard patient instruction; APR: adequate preparation rate; A: additional explanation; B: visual aid; C: newly designed booklet; D: new visual aids; E: SMS text messaging; F: telephone call; G: educational video; H: social media application; I: mobile app.

We did not find evidence of publication bias based on the funnel plot asymmetry in Figure 3, although the number of studies included in each comparison was very small, thereby making the available methods for evaluating publication bias somewhat unreliable. There were no significant differences between direct and indirect estimates where both were available, and the 2 methods had overlapping CIs for all interventions, as observed in Table 2.

## Discussion

Several meta-analyses [13,14,16-18] examined the quality of BP, colonic outcomes, and AEs including abdominal discomfort, nausea and vomiting, and sleep disturbance by comparing EPIs and SPIs. They concluded that EPIs are more effective and safer techniques for improving the quality of BP before colonoscopy. However, they did not completely explain which instruction is superior among the various EPIs because their analyses were based solely on direct comparisons between 2 given techniques. Our network meta-analysis of 23 RCTs involving 7969 patients is the first study that comprehensively analyzes direct and indirect evidence in EPIs for BP prior to colonoscopy.

In this systematic review and network meta-analysis, we made several key observations: (1) SMS text messaging and social media applications increase the APR, with high confidence in the estimates; additional explanations and telephone calls improve the APR with moderate confidence in the estimates; educational videos, newly designed booklets, and new visual aids also increase the APR but with low-to-very-low confidence in the estimates. (2) Based on high-to-moderate-quality evidence, additional explanations offer significant advantages over visual aids, telephone calls, and educational videos, but not SMS text messaging and social media applications; additional explanations are also superior to mobile apps but not to newly designed booklets and new visual aids, although the quality of evidence ranges from low to very low. (3) SMS text messaging, telephone calls, social media applications, and educational videos offer significant advantages over visual aids for increasing the APR, based on moderate-to-high-quality evidence; newly designed booklets also increase the APR with low-quality evidence. (4) According to low-quality evidence, newly designed booklets, SMS text messaging, educational videos, and social media applications significantly increase the APR but not new visual aids and telephone calls when compared to mobile apps. Overall, we observed that newly designed booklets had the highest probability of being ranked the best (for increasing APR), followed by additional explanations, new visual aids, social media applications, educational videos, SMS text messaging, telephone calls, and visual aids. (5) SMS text messaging, telephone calls, and social media applications increase adherence to and satisfaction with the BP regime; social media applications are associated with decreased risk of AEs; telephone calls or social media applications increase the PDR. It should be noted that we analyzed an inadequate number of eligible studies, and additional studies are warranted.

The quality of BP is the metric to determine if colonoscopy is successful and safe [63]. In this regard, 2 studies investigated the comparative efficacy of telephone calls and social media applications (WeChat) compared to SMS text messaging for BP and colonic outcomes. Lee and colleagues found no significant difference in the quality of BP between the telephone call and SMS text messaging groups, which is consistent with our finding (OR 0.82, 95% CI, 0.48-1.31; moderate quality evidence). However, the finding of Wang et al conflicts with the finding in the present study (OR 1.07, 95% CI 0.67-1.62; high quality of evidence), wherein social media applications are superior to SMS text messaging in increasing the APR. As a critically important quality metric of colonoscopy [63,64], the PDR was also assessed in these 2 studies, where no differences between the telephone call or social media application, and SMS text messaging groups were detected. However, our study demonstrates that telephone calls are associated with an increased PDR when compared to SMS text messaging (OR 1.71, 95% CI 1.2-2.36). Moreover, only 1 study with a small sample size was performed to compare telephone calls or social media applications to SMS text messaging. However, more studies performing indirect comparisons were included in our study to calculate the combined estimates. Further, this finding was supported by moderate- or high-quality evidence in our study.

The strengths of our analyses include the comprehensive and simultaneous assessment of the relative efficacy of all EPIs for BP prior to colonoscopy. Given the limited comparative effectiveness studies, it remains difficult for patients and physicians to make informed decisions about which instructions are most effective for improving the quality of BP. We used the GRADE methodology [34,35] to assess the quality of evidence for this network meta-analysis, which can be directly applied in guideline development.

However, there are certain limitations associated with direct comparative effectiveness related to network analyses and individual studies, which merit further discussion. First, there is a paucity of studies. Second, network meta-analysis may be vulnerable to misinterpretation. The biggest threat to the validity of network meta-analysis is conceptual heterogeneity, wherein there are considerable differences among the participants, interventions, background treatments, and outcome assessments, thus limiting the comparability of trials. It assumes that patients enrolled in all the included studies could have been sampled from the same theoretical population [20,35]. Although there were subtle differences in the patients (proportion of patients having undergone colonoscopy previously and those undergoing screening colonoscopy, diagnostic colonoscopy, or surveillance colonoscopy), BP solutions (such as polyethylene glycol [PEG] alone, PEG plus prokinetic agent, and low or standard doses), solution administration methods (split or single dose), and dietary restrictions (low residue, clear liquid, or low fiber), we tried to minimize this conceptual heterogeneity by performing multiple sensitivity analyses, including excluding trials using uncommon preparation assessment scales with high risk or multicenter design; the overall findings were unchanged, suggesting the robustness of these analyses. Third, the ranking probabilities may be challenging to interpret and do not always imply a clinically important difference. However, instead of focusing only on the summaries of the effect estimates, we used GRADE to rate the overall quality of evidence considering the risk of bias, imprecision, indirectness, inconsistency, and other biases for rating the confidence in the estimates [35].

There were similar limitations in the individual studies, which also undermine the strength of the meta-analysis. Most studies focused on the quality of BP, with a limited number of studies on colonic outcomes. BP-related AEs were poorly reported, limiting the assessments regarding the benefits of instructions; hence, a thorough assessment of risk-benefit profiles could not be performed. Studies were also at risk of detection bias with suboptimal reporting of the blinding to assessor outcomes. Various dietary restrictions were imposed in different eligible studies; however, previous meta-analyses have demonstrated no difference between low residues and clear liquids [12,65], and thus, sensitivity analysis was not designed according to these aspects. Although BP solutions with different doses were used in the included studies, we did not design the subgroup or sensitivity analysis according to the dose of the BP solution because no significant difference between low and traditional doses was confirmed in our meta-analysis [6]. Split doses proved beneficial compared to single doses for improving BP [66] and increasing the PDR and ADR [67]; however, the insufficient number of eligible studies poses limitations for designing further sensitivity analyses. Moreover, 5 BP assessment scales were used in all the eligible studies; thus, we performed a sensitivity analysis to examine the robustness of the pooled results through excluding studies in which uncommon scales were used and found that most of the results were unchanged.

Despite these limitations, our network meta-analysis provides a better understanding regarding the comparative efficacy of EPIs for BP prior to colonoscopy. Newly designed booklets, telephone calls, educational videos, and social media applications can increase the quality of BP. Telephone calls or social media applications may be associated with improved adherence to and satisfaction with the BP regime, decreased risk of AEs, or an increased PDR.

## Appendix

Multimedia Appendix 1 Full search strategy.

Multimedia Appendix 2 Classification and comparison of all types of enhanced patient instructions included in this study.

Multimedia Appendix 3 Characteristics of eligible studies.

Multimedia Appendix 4 All supplementary figures supporting our findings.

Multimedia Appendix 5 All sensitivity analyses according to predesigned criteria.

## Abbreviations

ABPS: Aronchick Bowel Preparation Scale
AEs: adverse events
AI: adherence to instruction
APR: adequate preparation rate
BBPS: Boston Bowel Preparation Scale
CENTRAL: Cochrane Central Register of Controlled Trials
EPIs: enhanced patient instructions
GRADE: Grading of Recommendations Assessment, Development and Evaluation
OBPS: Ottawa Bowel Preparation Scale
OR: odds ratio
PDR: polyp detection rate
PRISMA: Preferred Reporting Items for Systematic Reviews and Meta-Analyses
RCT: randomized controlled trial
SPIs: standard patient instructions

## References

[1] Bray F, Ferlay J, Soerjomataram I, Siegel RL, Torre LA, Jemal A (2018). Global cancer statistics 2018: GLOBOCAN estimates of incidence and mortality worldwide for 36 cancers in 185 countries. CA Cancer J Clin.

[2] Imperiale TF, Glowinski EA, Lin-Cooper C, Larkin GN, Rogge JD, Ransohoff DF (2008). Five-year risk of colorectal neoplasia after negative screening colonoscopy. N Engl J Med.

[3] Winawer SJ, Zauber AG, Ho MN, O'Brien MJ, Gottlieb LS, Sternberg SS, Waye JD, Schapiro M, Bond JH, Panish JF, Ackroyd F, Shike M, Kurtz RC, Hornsby-Lewis L, Gerdes H, Stewart ET (1993). Prevention of colorectal cancer by colonoscopic polypectomy. N Engl J Med.

[4] Zauber AG, Winawer SJ, O'Brien MJ, Lansdorp-Vogelaar I, van Ballegooijen M, Hankey BF, Shi W, Bond JH, Schapiro M, Panish JF, Stewart ET, Waye JD (2012). Colonoscopic polypectomy and long-term prevention of colorectal-cancer deaths. N Engl J Med.

[5] Zarchy TM, Ershoff D (1996). Risk of colorectal cancer in families of patients with adenomatous polyps. N Engl J Med.

[6] Yi L, Tian X, Shi B, Chen H, Liu X, Pi Y, Chen W (2019). Low-volume polyethylene glycol improved patient attendance in bowel preparation before colonoscopy: a meta-analysis with trial sequential analysis. Front Med.

[7] Sherer EA, Imler TD, Imperiale TF (2012). The effect of colonoscopy preparation quality on adenoma detection rates. Gastrointest Endosc.

[8] Tian X, Shi B, Chen H, Liu X, Tang R, Pi Y, Chen W (2019). Comparative efficacy of 2 L polyethylene glycol alone or with ascorbic acid vs. 4 L polyethylene glycol for colonoscopy: a systematic review and network meta-analysis of 12 randomized controlled trials. Front Med (Lausanne).

[9] Tian X, Shi B, Liu X, Chen H, Chen W (2019). A randomized trial of split dose 3 L polyethylene glycol lavage solution, 2 L polyethylene glycol lavage combined with castor oil, and 1 L of polyethylene glycol lavage solution combined with castor Oil and ascorbic acid for preparation for colonoscopy. Front Med (Lausanne).

[10] Rex D, Imperiale TF, Latinovich DR, Bratcher LL (2002). Impact of bowel preparation on efficiency and cost of colonoscopy. Am J Gastroenterol.

[11] Harewood GC, Sharma VK, de Garmo P (2003). Impact of colonoscopy preparation quality on detection of suspected colonic neoplasia. Gastrointest Endosc.

[12] Song G, Tian X, Ma L, Yi L, Shuai T, Zeng Z, Zeng X (2016). Regime for bowel preparation in patients scheduled to colonoscopy: low-residue diet or clear liquid diet? Evidence From Systematic Review With Power Analysis. Medicine (Baltimore).

[13] Kurlander JE, Sondhi AR, Waljee AK, Menees SB, Connell CM, Schoenfeld PS, Saini SD (2016). How efficacious are patient education interventions to improve bowel preparation for colonoscopy? a systematic review. PLoS One.

[14] Guo X, Yang Z, Zhao L, Leung F, Luo H, Kang X, Li X, Jia H, Yang S, Tao Q, Pan Y, Guo X (2017). Enhanced instructions improve the quality of bowel preparation for colonoscopy: a meta-analysis of randomized controlled trials. Gastrointest Endosc.

[15] Ness RM, Manam R, Hoen H, Chalasani N (2001). Predictors of inadequate bowel preparation for colonoscopy. Am J Gastroenterol.

[16] Chang C, Shih S, Wang H, Chu C, Wang T, Hung C, Shieh T, Lin Y, Chen M (2015). Meta-analysis: the effect of patient education on bowel preparation for colonoscopy. Endosc Int Open.

[17] Desai M, Nutalapati V, Bansal A, Buckles D, Bonino J, Olyaee M, Rastogi A (2019). Use of smartphone applications to improve quality of bowel preparation for colonoscopy: a systematic review and meta-analysis. Endosc Int Open.

[18] Gkolfakis P, Tziatzios G, Papanikolaou IS, Triantafyllou K (2019). Strategies to improve inpatients' quality of bowel preparation for colonoscopy: a systematic review and meta-analysis. Gastroenterol Res Pract.

[19] Tian X, Xu L, Liu X, Chen W (2020). Enhanced patient education for colonic polyp and adenoma detection: meta-analysis of randomized controlled trials. JMIR Mhealth Uhealth.

[20] Cipriani A, Higgins JP, Geddes JR, Salanti G (2013). Conceptual and technical challenges in network meta-analysis. Ann Intern Med.

[21] Mills EJ, Ioannidis JPA, Thorlund K, Schünemann HJ, Puhan MA, Guyatt GH (2012). How to use an article reporting a multiple treatment comparison meta-analysis. JAMA.

[22] Moher D, Liberati A, Tetzlaff J, Altman DG, PRISMA Group (2009). Preferred reporting items for systematic reviews and meta-analyses: the PRISMA statement. Ann Intern Med.

[23] Jansen JP, Fleurence R, Devine B, Itzler R, Barrett A, Hawkins N, Lee K, Boersma C, Annemans L, Cappelleri JC (2011). Interpreting indirect treatment comparisons and network meta-analysis for health-care decision making: report of the ISPOR Task Force on Indirect Treatment Comparisons Good Research Practices: part 1. Value Health.

[24] Higgins JPT, Altman DG, Gøtzsche PC, Jüni P, Moher D, Oxman AD, Savovic J, Schulz KF, Weeks L, Sterne JAC, Cochrane Bias Methods Group, Cochrane Statistical Methods Group (2011). The Cochrane Collaboration's tool for assessing risk of bias in randomised trials. BMJ.

[25] DerSimonian R, Laird N (1986). Meta-analysis in clinical trials. Control Clin Trials.

[26] Higgins JPT, Thompson SG, Deeks JJ, Altman DG (2003). Measuring inconsistency in meta-analyses. BMJ.

[27] Higgins JPT, Thomas J, Chandler J, Cumpston M, Li T, Page MJ, Welch VA (2021). Cochrane Handbook for Systematic Reviews of Interventions version 6.2.

[28] Page MJ, McKenzie JE, Higgins JPT (2018). Tools for assessing risk of reporting biases in studies and syntheses of studies: a systematic review. BMJ Open.

[29] Dias S, Sutton AJ, Ades AE, Welton NJ (2012). Evidence synthesis for decision making 2. Med Decis Making.

[30] Lu G, Ades AE (2004). Combination of direct and indirect evidence in mixed treatment comparisons. Statist Med.

[31] Sutton A, Ades AE, Cooper N, Abrams K (2008). Use of indirect and mixed treatment comparisons for technology assessment. Pharmacoeconomics.

[32] Song G, Liu X, Bian W, Wu J, Deng Y, Zhang H, Tian X (2017). Systematic review with network meta-analysis: comparative efficacy of different enteral immunonutrition formulas in patients underwent gastrectomy. Oncotarget.

[33] Singh S, Murad MH, Chandar AK, Bongiorno CM, Singal AK, Atkinson SR, Thursz MR, Loomba R, Shah VH (2015). Comparative effectiveness of pharmacological interventions for severe alcoholic hepatitis: a systematic review and network meta-analysis. Gastroenterology.

[34] Guyatt G, Oxman AD, Sultan S, Brozek J, Glasziou P, Alonso-Coello P, Atkins D, Kunz R, Montori V, Jaeschke R, Rind D, Dahm P, Akl EA, Meerpohl J, Vist G, Berliner E, Norris S, Falck-Ytter Y, Schünemann HJ (2013). GRADE guidelines: 11. making an overall rating of confidence in effect estimates for a single outcome and for all outcomes. J Clin Epidemiol.

[35] Puhan MA, Schünemann HJ, Murad MH, Li T, Brignardello-Petersen R, Singh JA, Kessels AG, Guyatt GH, GRADE Working Group (2014). A GRADE working group approach for rating the quality of treatment effect estimates from network meta-analysis. BMJ.

[36] Back SY, Kim HG, Ahn EM, Park S, Jeon SR, Im HH, Kim J, Ko BM, Lee JS, Lee TH, Cho J (2018). Impact of patient audiovisual re-education via a smartphone on the quality of bowel preparation before colonoscopy: a single-blinded randomized study. Gastrointest Endosc.

[37] Elvas L, Brito D, Areia M, Carvalho R, Alves S, Saraiva S, Cadime AT (2017). Impact of personalised patient education on bowel preparation for colonoscopy: prospective randomised controlled trial. GE Port J Gastroenterol.

[38] Garg S, Girotra M, Chandra L, Verma V, Kaur S, Allawy A, Secco A, Anand R, Dutta SK (2016). Improved bowel preparation with multimedia education in a predominantly African-American population: a randomized study. Diagn Ther Endosc.

[39] Jeon SC, Kim JH, Kim SJ, Kwon HJ, Choi YJ, Jung K, Kim SE, Moon W, Park MI, Park SJ (2019). Effect of sending educational video clips via smartphone mobile messenger on bowel preparation before colonoscopy. Clin Endosc.

[40] Kang X, Zhao L, Leung F, Luo H, Wang L, Wu J, Guo X, Wang X, Zhang L, Hui N, Tao Q, Jia H, Liu Z, Chen Z, Liu J, Wu K, Fan D, Pan Y, Guo X (2016). Delivery of instructions via mobile social media app increases quality of bowel preparation. Clin Gastroenterol Hepatol.

[41] Lee Y, Kim E, Choi J, Lee K, Park K, Cho K, Jang B, Chung W, Hwang J (2015). Impact of reinforced education by telephone and short message service on the quality of bowel preparation: a randomized controlled study. Endoscopy.

[42] Liu C, Song X, Hao H (2018). Educational video followed by retelling bowel preparation process to improve colonoscopy bowel preparation quality: a prospective nursing intervention study. Med Sci Monit.

[43] Liu X, Luo H, Zhang L, Leung FW, Liu Z, Wang X, Huang R, Hui N, Wu K, Fan D, Pan Y, Guo X (2014). Telephone-based re-education on the day before colonoscopy improves the quality of bowel preparation and the polyp detection rate: a prospective, colonoscopist-blinded, randomised, controlled study. Gut.

[44] Lorenzo-Zúñiga V, Moreno de Vega V, Marín I, Barberá M, Boix J (2015). Improving the quality of colonoscopy bowel preparation using a smart phone application: a randomized trial. Dig Endosc.

[45] Meng X (2015). Individualized health education improves bowel preparation before colonoscopy. WCJD.

[46] Modi C, Depasquale JR, Digiacomo WS, Malinowski JE, Engelhardt K, Shaikh SN, Kothari ST, Kottam R, Shakov R, Maksoud C, Baddoura WJ, Spira RS (2009). Impact of patient education on quality of bowel preparation in outpatient colonoscopies. Qual Prim Care.

[47] Park J, Kim MS, Kim H, Kim SI, Shin CH, Lee HJ, Lee WS, Moon S (2016). A randomized controlled trial of an educational video to improve quality of bowel preparation for colonoscopy. BMC Gastroenterol.

[48] Pillai A, Menon R, Oustecky D, Ahmad A (2018). Educational colonoscopy video enhances bowel preparation quality and comprehension in an inner city population. J Clin Gastroenterol.

[49] Prakash SR, Verma S, McGowan J, Smith BE, Shroff A, Gibson GH, Cheng M, Lowe Ii D, Gopal K, Mohanty SR (2013). Improving the quality of colonoscopy bowel preparation using an educational video. Can J Gastroenterol.

[50] Rice SC, Higginbotham T, Dean MJ, Slaughter JC, Yachimski PS, Obstein KL (2016). Video on diet before outpatient colonoscopy does not improve quality of bowel preparation: a prospective, randomized, controlled trial. Am J Gastroenterol.

[51] Sharara AI, Chalhoub JM, Beydoun M, Shayto RH, Chehab H, Harb AH, Mourad FH, Sarkis FS (2017). A customized mobile application in colonoscopy preparation: a randomized controlled trial. Clin Transl Gastroenterol.

[52] Spiegel BMR, Talley J, Shekelle P, Agarwal N, Snyder B, Bolus R, Kurzbard N, Chan M, Ho A, Kaneshiro M, Cordasco K, Cohen H (2011). Development and validation of a novel patient educational booklet to enhance colonoscopy preparation. Am J Gastroenterol.

[53] Tae JW, Lee JC, Hong SJ, Han JP, Lee YH, Chung JH, Yoon HG, Ko BM, Cho JY, Lee JS, Lee MS (2012). Impact of patient education with cartoon visual aids on the quality of bowel preparation for colonoscopy. Gastrointest Endosc.

[54] Walter B, Klare P, Strehle K, Aschenbeck J, Ludwig L, Dikopoulos N, Mayr M, Neu B, Hann A, Mayer B, Meining A, von Delius S (2019). Improving the quality and acceptance of colonoscopy preparation by reinforced patient education with short message service: results from a randomized, multicenter study (PERICLES-II). Gastrointest Endosc.

[55] Wang S, Wang Q, Yao J, Zhao S, Wang L, Li Z, Bai Y (2019). Effect of WeChat and short message service on bowel preparation. Eur J Gastroenterol Hepatol.

[56] Zhang Q, Li J, Zhang Q, Li Y, Lei C, Shang B, Guan X, Zhang Q (2018). Effect of education by messaging software on the quality of bowel preparation for colonoscopy. Chin Med J (Engl).

[57] Calderwood AH, Lai EJ, Fix OK, Jacobson BC (2011). An endoscopist-blinded, randomized, controlled trial of a simple visual aid to improve bowel preparation for screening colonoscopy. Gastrointest Endosc.

[58] Cho YY, Kim HO (2015). Effects of a patient educational video program on bowel preparation prior to colonoscopy. J Korean Acad Nurs.

[59] Ergen WF, Pasricha T, Hubbard FJ, Higginbotham T, Givens T, Slaughter JC, Obstein KL (2016). Providing hospitalized patients with an educational booklet increases the quality of colonoscopy bowel preparation. Clin Gastroenterol Hepatol.

[60] Hsu W, Liang C, Lin C, Lee T, Chung C (2016). A modified bowel preparation protocol improves the quality of bowel cleansing for colonoscopy. Advances in Digestive Medicine.

[61] Kakkar A, Jacobson BC (2013). Failure of an internet-based health care intervention for colonoscopy preparation: a caveat for investigators. JAMA Intern Med.

[62] Rosenfeld G, Krygier D, Enns RA, Singham J, Wiesinger H, Bressler B (2010). The impact of patient education on the quality of inpatient bowel preparation for colonoscopy. Can J Gastroenterol.

[63] Kaminski MF, Regula J, Kraszewska E, Polkowski M, Wojciechowska U, Didkowska J, Zwierko M, Rupinski M, Nowacki MP, Butruk E (2010). Quality indicators for colonoscopy and the risk of interval cancer. N Engl J Med.

[64] Corley DA, Jensen CD, Marks AR, Zhao WK, Lee JK, Doubeni CA, Zauber AG, de BJ, Fireman BH, Schottinger JE, Quinn VP, Ghai NR, Levin TR, Quesenberry CP (2014). Adenoma detection rate and risk of colorectal cancer and death. N Engl J Med.

[65] Nguyen DL, Jamal MM, Nguyen ET, Puli SR, Bechtold ML (2016). Low-residue versus clear liquid diet before colonoscopy: a meta-analysis of randomized, controlled trials. Gastrointest Endosc.

[66] Martel M, Barkun AN, Menard C, Restellini S, Kherad O, Vanasse A (2015). Split-dose preparations are superior to day-before bowel cleansing regimens: a meta-analysis. Gastroenterology.

[67] Zawaly K, Rumbolt C, Abou-Setta AM, Neilson C, Rabbani R, Zarychanski R, Singh H (2019). The efficacy of split-dose bowel preparations for polyp detection: a systematic review and meta-analysis. Am J Gastroenterol.

