# Supplementary material for: Comparative Effectiveness of Enhanced Patient Instructions for Bowel Preparation Before Colonoscopy: Network Meta-analysis of 23 Randomized Controlled Trials
Source: J Med Internet Res. 2021 Oct 25;23(10):e19915. doi: 10.2196/19915 (PMC8576559; doi:10.2196/19915)
Supplement: Multimedia Appendix 1 [file jmir_v23i10e19915_app1.docx]

**PubMed search strings**

| **Query** | **Results** |
| --- | --- |
| ((((((((((((((((((((("Education"[Mesh]) OR "education" [Subheading]) OR "Teaching"[Mesh]) OR "Patient Education as Topic"[Mesh]) OR "Pamphlets"[Mesh]) OR "Computer-Assisted Instruction"[Mesh]) OR "Teaching Materials"[Mesh]) OR "Video Recording"[Mesh]) OR "Audiovisual Aids"[Mesh]) OR "Mobile Applications"[Mesh]) OR "Smartphone"[Mesh]) OR "Telemedicine"[Mesh]) OR "Text Messaging"[Mesh]) OR "Internet"[Mesh]) OR "Software"[Mesh]) OR "Electronic Mail"[Mesh]) OR (((((((((((((((((((((((((((((((((((((((((((((((((((((((((((((((Educat*[Title/Abstract]) OR (Workshop*[Title/Abstract])) OR (Training Program*[Title/Abstract])) OR (Educational Activit*[Title/Abstract])) OR (Literacy Program*[Title/Abstract])) OR (Training Technique*[Title/Abstract])) OR (Training Technic*[Title/Abstract])) OR (Pedagog*[Title/Abstract])) OR (Teaching Method*[Title/Abstract])) OR (Academic Training[Title/Abstract])) OR (Training Activit*[Title/Abstract])) OR (Educational Technic*[Title/Abstract])) OR (Educational Technique*[Title/Abstract])) OR (Patient Education[Title/Abstract])) OR (Education of Patients[Title/Abstract])) OR (Pamphlet*[Title/Abstract])) OR (Brochure*[Title/Abstract])) OR (Booklet*[Title/Abstract])) OR (Computer Assisted Instruction*[Title/Abstract])) OR (Computer-Assisted Instruction*[Title/Abstract])) OR (Computerized Self-Instruction Program*[Title/Abstract])) OR (Computerized Programmed Instruction*[Title/Abstract])) OR (Teaching Material*[Title/Abstract])) OR (Video Recording*[Title/Abstract])) OR (Videorecording*[Title/Abstract])) OR (Audiovisual Recording*[Title/Abstract])) OR (Audiovisual Aid*[Title/Abstract])) OR (Audio-Visual Aid*[Title/Abstract])) OR (Audio Visual Aid*[Title/Abstract])) OR (Visual Aid*[Title/Abstract])) OR (Mobile Application*[Title/Abstract])) OR (Mobile App*[Title/Abstract])) OR (Portable Electronic App*[Title/Abstract])) OR (Portable Electronic Application*[Title/Abstract])) OR (Portable Software App*[Title/Abstract])) OR (Portable Software Application*[Title/Abstract])) OR (Smartphone*[Title/Abstract])) OR (Smart Phone*[Title/Abstract])) OR (Telemedicine[Title/Abstract])) OR (Mobile Health[Title/Abstract])) OR (mHealth[Title/Abstract])) OR (Telehealth[Title/Abstract])) OR (eHealth[Title/Abstract])) OR (Text Messaging[Title/Abstract])) OR (Texting*[Title/Abstract])) OR (Short Message Service[Title/Abstract])) OR (Text Message*[Title/Abstract])) OR (World Wide Web[Title/Abstract])) OR (Internet[Title/Abstract])) OR (Cyberspace[Title/Abstract])) OR (Cyber Space[Title/Abstract])) OR (Software[Title/Abstract])) OR (Computer Software[Title/Abstract])) OR (Computer Program*[Title/Abstract])) OR (Software Tool*[Title/Abstract])) OR (Software Engineering[Title/Abstract])) OR (Computer Applications Software*[Title/Abstract])) OR (Computer Software Application*[Title/Abstract])) OR (Computer Programs[Title/Abstract] AND Programming[Title/Abstract])) OR (Electronic Mail[Title/Abstract])) OR (Email*[Title/Abstract])) OR (E-Mail*[Title/Abstract])) OR (E Mail[Title/Abstract]))) AND (("Colonoscopy"[Mesh]) OR (((Colonoscop*[Title/Abstract]) OR (Colonoscopic Surgical Procedure*[Title/Abstract])) OR (Colonoscopic Surger*[Title/Abstract])))) AND ((("Cathartics"[Mesh]) OR "Cathartics" [Pharmacological Action]) OR (((((bowel preparation[Title/Abstract]) OR (bowel cleansing[Title/Abstract])) OR (bowel evacuant*[Title/Abstract])) OR (purgative*[Title/Abstract])) OR (Bowel Preparation Solution*[Title/Abstract])))) AND (((((((("Randomized Controlled Trial" [Publication Type]) OR "Randomized Controlled Trials as Topic"[Mesh]) OR "Random Allocation"[Mesh]) OR "Double-Blind Method"[Mesh]) OR "Single-Blind Method"[Mesh]) OR "Placebos"[Mesh]) OR ((random*[Text Word]) OR (placebo[Title/Abstract]))) OR (((((singl*[Text Word]) OR (doubl*[Text Word])) OR (trebl*[Text Word])) OR (tripl*[Text Word])) AND (((mask*[Text Word]) OR (blind*[Text Word])) OR (dumm*[Text Word]))))) AND (("1950"[Date - Publication] : "2020/02/29"[Date - Publication]))) AND (English[Language]) | 85 |
| English[Language] | 26,876,547 |
| ("1950"[Date - Publication] : "2020/02/29"[Date - Publication]) | 30,175,556 |
| ((((((((((((((((((("Education"[Mesh]) OR "education" [Subheading]) OR "Teaching"[Mesh]) OR "Patient Education as Topic"[Mesh]) OR "Pamphlets"[Mesh]) OR "Computer-Assisted Instruction"[Mesh]) OR "Teaching Materials"[Mesh]) OR "Video Recording"[Mesh]) OR "Audiovisual Aids"[Mesh]) OR "Mobile Applications"[Mesh]) OR "Smartphone"[Mesh]) OR "Telemedicine"[Mesh]) OR "Text Messaging"[Mesh]) OR "Internet"[Mesh]) OR "Software"[Mesh]) OR "Electronic Mail"[Mesh]) OR (((((((((((((((((((((((((((((((((((((((((((((((((((((((((((((((Educat*[Title/Abstract]) OR (Workshop*[Title/Abstract])) OR (Training Program*[Title/Abstract])) OR (Educational Activit*[Title/Abstract])) OR (Literacy Program*[Title/Abstract])) OR (Training Technique*[Title/Abstract])) OR (Training Technic*[Title/Abstract])) OR (Pedagog*[Title/Abstract])) OR (Teaching Method*[Title/Abstract])) OR (Academic Training[Title/Abstract])) OR (Training Activit*[Title/Abstract])) OR (Educational Technic*[Title/Abstract])) OR (Educational Technique*[Title/Abstract])) OR (Patient Education[Title/Abstract])) OR (Education of Patients[Title/Abstract])) OR (Pamphlet*[Title/Abstract])) OR (Brochure*[Title/Abstract])) OR (Booklet*[Title/Abstract])) OR (Computer Assisted Instruction*[Title/Abstract])) OR (Computer-Assisted Instruction*[Title/Abstract])) OR (Computerized Self-Instruction Program*[Title/Abstract])) OR (Computerized Programmed Instruction*[Title/Abstract])) OR (Teaching Material*[Title/Abstract])) OR (Video Recording*[Title/Abstract])) OR (Videorecording*[Title/Abstract])) OR (Audiovisual Recording*[Title/Abstract])) OR (Audiovisual Aid*[Title/Abstract])) OR (Audio-Visual Aid*[Title/Abstract])) OR (Audio Visual Aid*[Title/Abstract])) OR (Visual Aid*[Title/Abstract])) OR (Mobile Application*[Title/Abstract])) OR (Mobile App*[Title/Abstract])) OR (Portable Electronic App*[Title/Abstract])) OR (Portable Electronic Application*[Title/Abstract])) OR (Portable Software App*[Title/Abstract])) OR (Portable Software Application*[Title/Abstract])) OR (Smartphone*[Title/Abstract])) OR (Smart Phone*[Title/Abstract])) OR (Telemedicine[Title/Abstract])) OR (Mobile Health[Title/Abstract])) OR (mHealth[Title/Abstract])) OR (Telehealth[Title/Abstract])) OR (eHealth[Title/Abstract])) OR (Text Messaging[Title/Abstract])) OR (Texting*[Title/Abstract])) OR (Short Message Service[Title/Abstract])) OR (Text Message*[Title/Abstract])) OR (World Wide Web[Title/Abstract])) OR (Internet[Title/Abstract])) OR (Cyberspace[Title/Abstract])) OR (Cyber Space[Title/Abstract])) OR (Software[Title/Abstract])) OR (Computer Software[Title/Abstract])) OR (Computer Program*[Title/Abstract])) OR (Software Tool*[Title/Abstract])) OR (Software Engineering[Title/Abstract])) OR (Computer Applications Software*[Title/Abstract])) OR (Computer Software Application*[Title/Abstract])) OR (Computer Programs[Title/Abstract] AND Programming[Title/Abstract])) OR (Electronic Mail[Title/Abstract])) OR (Email*[Title/Abstract])) OR (E-Mail*[Title/Abstract])) OR (E Mail[Title/Abstract]))) AND (("Colonoscopy"[Mesh]) OR (((Colonoscop*[Title/Abstract]) OR (Colonoscopic Surgical Procedure*[Title/Abstract])) OR (Colonoscopic Surger*[Title/Abstract])))) AND ((("Cathartics"[Mesh]) OR "Cathartics" [Pharmacological Action]) OR (((((bowel preparation[Title/Abstract]) OR (bowel cleansing[Title/Abstract])) OR (bowel evacuant*[Title/Abstract])) OR (purgative*[Title/Abstract])) OR (Bowel Preparation Solution*[Title/Abstract])))) AND (((((((("Randomized Controlled Trial" [Publication Type]) OR "Randomized Controlled Trials as Topic"[Mesh]) OR "Random Allocation"[Mesh]) OR "Double-Blind Method"[Mesh]) OR "Single-Blind Method"[Mesh]) OR "Placebos"[Mesh]) OR ((random*[Text Word]) OR (placebo[Title/Abstract]))) OR (((((singl*[Text Word]) OR (doubl*[Text Word])) OR (trebl*[Text Word])) OR (tripl*[Text Word])) AND (((mask*[Text Word]) OR (blind*[Text Word])) OR (dumm*[Text Word])))) | 101 |
| ((((((("Randomized Controlled Trial" [Publication Type]) OR "Randomized Controlled Trials as Topic"[Mesh]) OR "Random Allocation"[Mesh]) OR "Double-Blind Method"[Mesh]) OR "Single-Blind Method"[Mesh]) OR "Placebos"[Mesh]) OR ((random*[Text Word]) OR (placebo[Title/Abstract]))) OR (((((singl*[Text Word]) OR (doubl*[Text Word])) OR (trebl*[Text Word])) OR (tripl*[Text Word])) AND (((mask*[Text Word]) OR (blind*[Text Word])) OR (dumm*[Text Word]))) | 1,507,126 |
| ((((singl*[Text Word]) OR (doubl*[Text Word])) OR (trebl*[Text Word])) OR (tripl*[Text Word])) AND (((mask*[Text Word]) OR (blind*[Text Word])) OR (dumm*[Text Word])) | 208,565 |
| ((mask*[Text Word]) OR (blind*[Text Word])) OR (dumm*[Text Word]) | 399,081 |
| (((singl*[Text Word]) OR (doubl*[Text Word])) OR (trebl*[Text Word])) OR (tripl*[Text Word]) | 2,432,823 |
| (random*[Text Word]) OR (placebo[Title/Abstract]) | 1,451,905 |
| ((((("Randomized Controlled Trial" [Publication Type]) OR "Randomized Controlled Trials as Topic"[Mesh]) OR "Random Allocation"[Mesh]) OR "Double-Blind Method"[Mesh]) OR "Single-Blind Method"[Mesh]) OR "Placebos"[Mesh] | 778,677 |
| (("Cathartics"[Mesh]) OR "Cathartics" [Pharmacological Action]) OR (((((bowel preparation[Title/Abstract]) OR (bowel cleansing[Title/Abstract])) OR (bowel evacuant*[Title/Abstract])) OR (purgative*[Title/Abstract])) OR (Bowel Preparation Solution*[Title/Abstract])) | 26,346 |
| ((((bowel preparation[Title/Abstract]) OR (bowel cleansing[Title/Abstract])) OR (bowel evacuant*[Title/Abstract])) OR (purgative*[Title/Abstract])) OR (Bowel Preparation Solution*[Title/Abstract]) | 4,447 |
| ("Cathartics"[Mesh]) OR "Cathartics" [Pharmacological Action] | 23,282 |
| ("Colonoscopy"[Mesh]) OR (((Colonoscop*[Title/Abstract]) OR (Colonoscopic Surgical Procedure*[Title/Abstract])) OR (Colonoscopic Surger*[Title/Abstract])) | 45,218 |
| ((Colonoscop*[Title/Abstract]) OR (Colonoscopic Surgical Procedure*[Title/Abstract])) OR (Colonoscopic Surger*[Title/Abstract]) | 30,887 |
| "Colonoscopy"[Mesh] | 30,362 |
| (((((((((((((((("Education"[Mesh]) OR "education" [Subheading]) OR "Teaching"[Mesh]) OR "Patient Education as Topic"[Mesh]) OR "Pamphlets"[Mesh]) OR "Computer-Assisted Instruction"[Mesh]) OR "Teaching Materials"[Mesh]) OR "Video Recording"[Mesh]) OR "Audiovisual Aids"[Mesh]) OR "Mobile Applications"[Mesh]) OR "Smartphone"[Mesh]) OR "Telemedicine"[Mesh]) OR "Text Messaging"[Mesh]) OR "Internet"[Mesh]) OR "Software"[Mesh]) OR "Electronic Mail"[Mesh]) OR (((((((((((((((((((((((((((((((((((((((((((((((((((((((((((((((Educat*[Title/Abstract]) OR (Workshop*[Title/Abstract])) OR (Training Program*[Title/Abstract])) OR (Educational Activit*[Title/Abstract])) OR (Literacy Program*[Title/Abstract])) OR (Training Technique*[Title/Abstract])) OR (Training Technic*[Title/Abstract])) OR (Pedagog*[Title/Abstract])) OR (Teaching Method*[Title/Abstract])) OR (Academic Training[Title/Abstract])) OR (Training Activit*[Title/Abstract])) OR (Educational Technic*[Title/Abstract])) OR (Educational Technique*[Title/Abstract])) OR (Patient Education[Title/Abstract])) OR (Education of Patients[Title/Abstract])) OR (Pamphlet*[Title/Abstract])) OR (Brochure*[Title/Abstract])) OR (Booklet*[Title/Abstract])) OR (Computer Assisted Instruction*[Title/Abstract])) OR (Computer-Assisted Instruction*[Title/Abstract])) OR (Computerized Self-Instruction Program*[Title/Abstract])) OR (Computerized Programmed Instruction*[Title/Abstract])) OR (Teaching Material*[Title/Abstract])) OR (Video Recording*[Title/Abstract])) OR (Videorecording*[Title/Abstract])) OR (Audiovisual Recording*[Title/Abstract])) OR (Audiovisual Aid*[Title/Abstract])) OR (Audio-Visual Aid*[Title/Abstract])) OR (Audio Visual Aid*[Title/Abstract])) OR (Visual Aid*[Title/Abstract])) OR (Mobile Application*[Title/Abstract])) OR (Mobile App*[Title/Abstract])) OR (Portable Electronic App*[Title/Abstract])) OR (Portable Electronic Application*[Title/Abstract])) OR (Portable Software App*[Title/Abstract])) OR (Portable Software Application*[Title/Abstract])) OR (Smartphone*[Title/Abstract])) OR (Smart Phone*[Title/Abstract])) OR (Telemedicine[Title/Abstract])) OR (Mobile Health[Title/Abstract])) OR (mHealth[Title/Abstract])) OR (Telehealth[Title/Abstract])) OR (eHealth[Title/Abstract])) OR (Text Messaging[Title/Abstract])) OR (Texting*[Title/Abstract])) OR (Short Message Service[Title/Abstract])) OR (Text Message*[Title/Abstract])) OR (World Wide Web[Title/Abstract])) OR (Internet[Title/Abstract])) OR (Cyberspace[Title/Abstract])) OR (Cyber Space[Title/Abstract])) OR (Software[Title/Abstract])) OR (Computer Software[Title/Abstract])) OR (Computer Program*[Title/Abstract])) OR (Software Tool*[Title/Abstract])) OR (Software Engineering[Title/Abstract])) OR (Computer Applications Software*[Title/Abstract])) OR (Computer Software Application*[Title/Abstract])) OR (Computer Programs[Title/Abstract] AND Programming[Title/Abstract])) OR (Electronic Mail[Title/Abstract])) OR (Email*[Title/Abstract])) OR (E-Mail*[Title/Abstract])) OR (E Mail[Title/Abstract])) | 1,795,391 |
| ((((((((((((((((((((((((((((((((((((((((((((((((((((((((((((((Educat*[Title/Abstract]) OR (Workshop*[Title/Abstract])) OR (Training Program*[Title/Abstract])) OR (Educational Activit*[Title/Abstract])) OR (Literacy Program*[Title/Abstract])) OR (Training Technique*[Title/Abstract])) OR (Training Technic*[Title/Abstract])) OR (Pedagog*[Title/Abstract])) OR (Teaching Method*[Title/Abstract])) OR (Academic Training[Title/Abstract])) OR (Training Activit*[Title/Abstract])) OR (Educational Technic*[Title/Abstract])) OR (Educational Technique*[Title/Abstract])) OR (Patient Education[Title/Abstract])) OR (Education of Patients[Title/Abstract])) OR (Pamphlet*[Title/Abstract])) OR (Brochure*[Title/Abstract])) OR (Booklet*[Title/Abstract])) OR (Computer Assisted Instruction*[Title/Abstract])) OR (Computer-Assisted Instruction*[Title/Abstract])) OR (Computerized Self-Instruction Program*[Title/Abstract])) OR (Computerized Programmed Instruction*[Title/Abstract])) OR (Teaching Material*[Title/Abstract])) OR (Video Recording*[Title/Abstract])) OR (Videorecording*[Title/Abstract])) OR (Audiovisual Recording*[Title/Abstract])) OR (Audiovisual Aid*[Title/Abstract])) OR (Audio-Visual Aid*[Title/Abstract])) OR (Audio Visual Aid*[Title/Abstract])) OR (Visual Aid*[Title/Abstract])) OR (Mobile Application*[Title/Abstract])) OR (Mobile App*[Title/Abstract])) OR (Portable Electronic App*[Title/Abstract])) OR (Portable Electronic Application*[Title/Abstract])) OR (Portable Software App*[Title/Abstract])) OR (Portable Software Application*[Title/Abstract])) OR (Smartphone*[Title/Abstract])) OR (Smart Phone*[Title/Abstract])) OR (Telemedicine[Title/Abstract])) OR (Mobile Health[Title/Abstract])) OR (mHealth[Title/Abstract])) OR (Telehealth[Title/Abstract])) OR (eHealth[Title/Abstract])) OR (Text Messaging[Title/Abstract])) OR (Texting*[Title/Abstract])) OR (Short Message Service[Title/Abstract])) OR (Text Message*[Title/Abstract])) OR (World Wide Web[Title/Abstract])) OR (Internet[Title/Abstract])) OR (Cyberspace[Title/Abstract])) OR (Cyber Space[Title/Abstract])) OR (Software[Title/Abstract])) OR (Computer Software[Title/Abstract])) OR (Computer Program*[Title/Abstract])) OR (Software Tool*[Title/Abstract])) OR (Software Engineering[Title/Abstract])) OR (Computer Applications Software*[Title/Abstract])) OR (Computer Software Application*[Title/Abstract])) OR (Computer Programs[Title/Abstract] AND Programming[Title/Abstract])) OR (Electronic Mail[Title/Abstract])) OR (Email*[Title/Abstract])) OR (E-Mail*[Title/Abstract])) OR (E Mail[Title/Abstract]) | 970,511 |
| ((((((((((((((("Education"[Mesh]) OR "education" [Subheading]) OR "Teaching"[Mesh]) OR "Patient Education as Topic"[Mesh]) OR "Pamphlets"[Mesh]) OR "Computer-Assisted Instruction"[Mesh]) OR "Teaching Materials"[Mesh]) OR "Video Recording"[Mesh]) OR "Audiovisual Aids"[Mesh]) OR "Mobile Applications"[Mesh]) OR "Smartphone"[Mesh]) OR "Telemedicine"[Mesh]) OR "Text Messaging"[Mesh]) OR "Internet"[Mesh]) OR "Software"[Mesh]) OR "Electronic Mail"[Mesh] | 1,223,865 |

**Cochrane Central Register of Controlled Trials (CENTRAL) search strings**

**ID Search Hits**

#1 ("educat*" OR "workshop*" OR "training program*" OR "educational activit*" OR "literacy program*" OR "training technique*" OR "training technic*" OR "pedagog*" OR "teaching method*" OR "academic training" OR "training activit*" OR "educational technic*" OR "educational technique*" OR "patient education" OR "education of patients" OR "pamphlet*" OR "brochure*" OR "booklet*" OR "computer assisted instruction*" OR "computer assisted instruction*" OR "computerized self instruction program*" OR "computerized programmed instruction*" OR "teaching material*" OR "video recording*" OR "videorecording*" OR "audiovisual recording*" OR "audiovisual aid*" OR "audio visual aid*" OR "audio visual aid*" OR "visual aid*" OR "mobile application*" OR "mobile app*" OR "portable electronic app*" OR "portable electronic application*" OR "portable software app*" OR "portable software application*" OR "smartphone*" OR "smart phone*" OR "Telemedicine" OR "mobile health" OR "mHealth" OR "Telehealth" OR "eHealth" OR "text messaging" OR "texting*" OR "short message service" OR "text message*" OR "world wide web" OR "Internet" OR "Cyberspace" OR "cyber space" OR "Software" OR "computer software" OR "computer program*" OR "software tool*" OR "software engineering" OR "computer applications software*" OR "computer software application*" OR ("computer programs" AND "Programming") OR "electronic mail" OR "email*" OR "e mail*" OR "e mail"):ti,ab,kw (Word variations have been searched) 90497

#2 MeSH descriptor: [Education] explode all trees 32406

#3 MeSH descriptor: [Teaching] explode all trees 4284

#4 MeSH descriptor: [Patient Education as Topic] explode all trees 8840

#5 MeSH descriptor: [Pamphlets] explode all trees 889

#6 MeSH descriptor: [Computer-Assisted Instruction] explode all trees 1214

#7 MeSH descriptor: [Teaching Materials] explode all trees 4241

#8 MeSH descriptor: [Video Recording] explode all trees 2576

#9 MeSH descriptor: [Audiovisual Aids] explode all trees 3742

#10 MeSH descriptor: [Mobile Applications] explode all trees 628

#11 MeSH descriptor: [Smartphone] explode all trees 384

#12 MeSH descriptor: [Telemedicine] explode all trees 2524

#13 MeSH descriptor: [Text Messaging] explode all trees 848

#14 MeSH descriptor: [Internet] explode all trees 3969

#15 MeSH descriptor: [Software] explode all trees 3387

#16 MeSH descriptor: [Electronic Mail] explode all trees 324

#17 #1 OR #2 OR #3 OR #4 OR #5 OR #6 OR #7 OR #8 OR #9 OR #10 OR #11 OR #12 OR #13 OR #14 OR #15 OR #16 109931

#18 (Colonoscop* OR Colonoscopic Surgical Procedure* OR Colonoscopic Surger*):ti,ab,kw 6759

#19 MeSH descriptor: [Colonoscopy] explode all trees 1990

#20 #18 OR #19 6955

#21 (bowel preparation OR bowel cleansing OR bowel evacuant* OR purgative* OR bowel preparation solution*):ti,ab,kw 2810

#22 MeSH descriptor: [Cathartics] explode all trees 737

#23 #21 OR #22 3137

#24 (random* OR placebo*):ti,ab,kw 1096887

#25 (singl* OR doubl* OR trebl* OR trip*):ti,ab,kw 471233

#26 (mask* OR blind* OR dumm*):ti,ab,kw 391244

#27 #25 AND #26 349277

#28 #24 OR #27 1129124

#29 MeSH descriptor: [Randomized Controlled Trial] explode all trees 118

#30 MeSH descriptor: [Randomized Controlled Trials as Topic] explode all trees 14512

#31 MeSH descriptor: [Random Allocation] explode all trees 20604

#32 MeSH descriptor: [Double-Blind Method] explode all trees 137713

#33 MeSH descriptor: [Single-Blind Method] explode all trees 20810

#34 MeSH descriptor: [Placebos] explode all trees 24036

#35 #28 OR #29 OR #30 OR #31 OR #31 OR #32 OR #33 OR #34 1129170

#36 #17 AND #20 AND #23 AND #35 188

**Embase search strings**

| **ID** | **Query** | **Results** |
| --- | --- | --- |
| #14 | #3 AND #6 AND #9 AND #12 AND [english]/lim AND [1-1-1900]/sd NOT [1-3-2020]/sd | **462** |
| #13 | #3 AND #6 AND #9 AND #12 | **513** |
| #12 | #10 OR #11 | **3542089** |
| #11 | 'randomized controlled trial (topic)'/exp OR 'equivalence trial'/exp OR 'randomization'/exp OR 'double blind procedure'/exp OR 'single blind procedure'/exp OR 'placebo'/exp OR 'phase 3 clinical trial'/exp OR 'pragmatic trial'/exp OR 'controlled clinical trial'/exp OR 'controlled clinical trial (topic)'/exp | **1300169** |
| #10 | 'randomized controlled trial':ab,ti AND topic:ab,ti OR 'randomized controlled trial topic':ab,ti OR ('controlled clinical trial':ab,ti AND topic:ab,ti) OR 'controlled clinical trial topic'/exp OR 'controlled clinical trial topic' OR 'randomization'/exp OR randomization OR 'random allocation'/exp OR 'random allocation' OR (random AND allocation) OR 'double-blind method'/exp OR 'double-blind method' OR ('double blind' AND ('method'/exp OR method)) OR 'double blind procedure'/exp OR 'double blind procedure' OR (double AND ('blind'/exp OR blind) AND ('procedure'/exp OR procedure)) OR 'double-blind studies'/exp OR 'double-blind studies' OR ('double blind' AND ('studies'/exp OR studies)) OR 'single-blind method'/exp OR 'single-blind method' OR ('single blind' AND ('method'/exp OR method)) OR 'single blind procedure'/exp OR 'single blind procedure' OR (single AND ('blind'/exp OR blind) AND ('procedure'/exp OR procedure)) OR 'single-blind studies'/exp OR 'single-blind studies' OR ('single blind' AND ('studies'/exp OR studies)) OR (('control'/exp OR control) AND group*) OR random* OR sham OR placebo* OR ((singl* OR doubl*) AND adj AND (blind* OR dumm* OR mask*)) OR ((tripl* OR trebl*) AND adj AND (blind* OR dumm* OR mask*)) OR (control* AND adj3 AND ('study'/exp OR study OR 'studies'/exp OR studies OR trial* OR group*)) OR ((nonrandom* OR non) AND random*) OR 'non random*' OR 'quasi random*' OR quasirandom* OR allocated OR ((open AND label OR 'open label') AND adj5 AND ('study'/exp OR study OR 'studies'/exp OR studies OR trial*)) OR ((equivalence OR superiority OR 'non inferiority' OR noninferiority) AND adj3 AND ('study'/exp OR study OR 'studies'/exp OR studies OR trial*)) OR 'pragmatic study' OR (pragmatic AND ('study'/exp OR study)) OR 'pragmatic studies' OR (pragmatic AND ('studies'/exp OR studies)) OR ((pragmatic OR practical) AND adj3 AND trial*) OR ((quasiexperimental OR 'quasi experimental') AND adj3 AND ('study'/exp OR study OR 'studies'/exp OR studies OR trial*)) OR 'phase adj3 adj3' OR (phase AND (iii OR '3') AND adj3 AND ('study'/exp OR study OR 'studies'/exp OR studies OR trial*)) | **3413936** |
| #9 | #7 OR #8 | **175824** |
| #8 | 'laxative'/exp OR 'intestine preparation'/exp | **173865** |
| #7 | 'bowel preparation':ab,ti OR 'bowel cleansing':ab,ti OR 'bowel evacuant*':ab,ti OR purgative*:ab,ti OR 'bowel preparation solution*':ab,ti | **8686** |
| #6 | #4 OR #5 | **90307** |
| #5 | 'colonoscopy'/exp | **81467** |
| #4 | colonoscop*:ab,ti OR 'colonoscopic surgical procedure*':ab,ti OR 'colonoscopic surger*':ab,ti | **59931** |
| #3 | #1 OR #2 | **2528513** |
| #2 | 'education'/exp OR 'patient education'/exp OR 'teaching'/exp OR 'videorecording'/exp OR 'audiovisual aid'/exp OR 'mobile application'/exp OR 'smartphone'/exp OR 'telemedicine'/exp OR 'text messaging'/exp OR 'internet'/exp OR 'software'/exp OR 'e-mail'/exp | **2005986** |
| #1 | educat*:ab,ti OR workshop*:ab,ti OR 'training program*':ab,ti OR 'educational activit*':ab,ti OR 'literacy program*':ab,ti OR 'training technique*':ab,ti OR 'training technic*':ab,ti OR pedagog*:ab,ti OR 'teaching method*':ab,ti OR 'academic training':ab,ti OR 'training activit*':ab,ti OR 'educational technic*':ab,ti OR 'educational technique*':ab,ti OR 'patient education':ab,ti OR 'education of patients':ab,ti OR pamphlet*:ab,ti OR brochure*:ab,ti OR booklet*:ab,ti OR 'computer assisted instruction*':ab,ti OR 'computerized self instruction program*':ab,ti OR 'computerized programmed instruction*':ab,ti OR 'teaching material*':ab,ti OR 'video recording*':ab,ti OR videorecording*:ab,ti OR 'audiovisual recording*':ab,ti OR 'audiovisual aid*':ab,ti OR 'audio visual aid*':ab,ti OR 'visual aid*':ab,ti OR 'mobile application*':ab,ti OR 'mobile app*':ab,ti OR 'portable electronic app*':ab,ti OR 'portable electronic application*':ab,ti OR 'portable software app*':ab,ti OR 'portable software application*':ab,ti OR smartphone*:ab,ti OR 'smart phone*':ab,ti OR telemedicine:ab,ti OR 'mobile health':ab,ti OR mhealth:ab,ti OR telehealth:ab,ti OR ehealth:ab,ti OR 'text messaging':ab,ti OR texting*:ab,ti OR 'short message service':ab,ti OR 'text message*':ab,ti OR 'world wide web':ab,ti OR internet:ab,ti OR cyberspace:ab,ti OR 'cyber space':ab,ti OR software:ab,ti OR 'computer software':ab,ti OR 'computer program*':ab,ti OR 'software tool*':ab,ti OR 'software engineering':ab,ti OR 'computer applications software*':ab,ti OR 'computer software application*':ab,ti OR 'computer programs':ab,ti OR programming:ab,ti OR 'electronic mail':ab,ti OR email*:ab,ti OR 'e mail*':ab,ti | **1356746** |
