# Supplementary material for: Comparative Effectiveness of Enhanced Patient Instructions for Bowel Preparation Before Colonoscopy: Network Meta-analysis of 23 Randomized Controlled Trials
Source: J Med Internet Res. 2021 Oct 25;23(10):e19915. doi: 10.2196/19915 (PMC8576559; doi:10.2196/19915)
Supplement: Multimedia Appendix 2 [file jmir_v23i10e19915_app2.docx]

**Supplementary Table 2.** Classification and comparison of EPI regimes in the included trials.

| **EPI regime** | **Description** | **Possible advantages compared with SPI** | | | |
| --- | --- | --- | --- | --- | --- |
|  |  | **Easy understanding** | **Easy accessing** | **More interactive** | **Augmenting memory** |
| **Additional explanation** | Reviewing the questionnaire completed by the patients, providing additional explanation to the identified incorrect knowledge associated with BP procedure. | Yes |  | Yes |  |
| **Visual aid** | Providing patients with several endoscopic images demonstrating poor and good bowel preparation and some colonic polyps and adenomas. | Yes |  |  |  |
| **New visual aids** | Providing cartoons with several pictures delineating poor and good bowel preparation, dangerous complications during colonoscopy and foods to avoid. | Yes |  |  |  |
| **Newly designed booklet** | Including an overview emphasizing the importance patient participation, steps of daily BP instructions before colonoscopy, pictures of allowable and prohibited food and a visual color scale for interpreting grade of effluent. | Yes |  |  |  |
| **SMS** | Sending repeated text-message to patients to remind the schedule colonoscopy appointment time, dietary restrictions, adequate timing of purgative intake and answering the questions. |  |  | Yes | Yes |
| **Mobile app** | Using a novel application to introduce all information related to the colonoscopy appointment time, improtance of bowel preparation quality, method of ingesting bowel-cleansing agents, dietary recommendations, possible adverse events during bowel preparation and colonoscopy etc. | Yes | Yes |  |  |
| **Phone call** | Calling patients to emphasize the importance of bowel preparation, the directions for use and side effects of purgatives, the proper food type, and the start time and encouraging patients to contact the investigator if they had any questions. |  |  | Yes | Yes |
| **Social media app** | Using social media app such as WeChat to provide information of ingesting purgatives, dietary restriction and possible adverse events and answering the questions. |  | Yes | Yes |  |
| **Educational video** | Sending educational video to introduce all information related to the colonoscopy appointment time, improtance of bowel preparation quality, method of ingesting bowel-cleansing agents, dietary recommendations, possible adverse events during bowel preparation and colonoscopy etc. | Yes | Yes |  |  |

EPI: enhanced patient instruction; SPI: standard patient instruction; SMS: short message service.
