# Supplementary material for: Comparative Effectiveness of Enhanced Patient Instructions for Bowel Preparation Before Colonoscopy: Network Meta-analysis of 23 Randomized Controlled Trials
Source: J Med Internet Res. 2021 Oct 25;23(10):e19915. doi: 10.2196/19915 (PMC8576559; doi:10.2196/19915)
Supplement: Multimedia Appendix 3 [file jmir_v23i10e19915_app3.docx]

| **Supplementary Table 3.** Characteristics of studies comparing different educational instructions for bowel preparation prior to colonoscopy. | | | | | | | | | |
| --- | --- | --- | --- | --- | --- | --- | --- | --- | --- |
| Study | Design | Comparison | BP regime | Administration method | Previous colonoscopy, % | Indications | Diet restriction | BP scale | Time of EPI |
| Back 2018 | Single center | Educational video vs SPI | 4L PEG, 2L PEG plus Asc, SPMC | Split dose | 38.1 vs 31.9 | Mixed colonoscopy | Clear liquid | BBPS | 1 day before |
| Calderwood 2011 | Single center | Visual aid vs SPI | 4L PEG alone or plus bisacodyl | n.r. | 15.5 vs 16.5 | Screening colonoscopy | n.r. | BBPS | n.r. |
| Cho 2015 | Single center | Educational video vs SPI | 2L PEG plus Asc | Single dose | 49.0 vs 42.0 | Screening colonoscopy | Clear liquid | ABPS | 3 days before |
| Elvas 2017 | Single center | Additional explanation vs SPI | 4L PEG | Single dose | 55.2 vs 71.7 | n.r. | Clear liquid | ABPS | n.r. |
| Garg 2016 | Single center | Educational video vs SPI | n.r. | Single dose | 35.4 vs 34.8 | Mixed colonoscopy | Clear liquid | ABPS | 1 day before |
| Jeon 2018 | Single center | Educational video vs SPI | 2L PEG | Split dose | 52.1 vs 58.9 | Screening colonoscopy | Low fiber | OBPS | 1 day before |
| Kang 2016 | Single center | Social media app vs SPI | 4L PEG | Split dose | 23.5 vs 29.2 | Mixed conoloscopy | Clear liquid | OBPS | 15 days before |
| Lee 2015 | Single center | SMS vs Phone call vs SPI | 2L PEG plus Asc | Split dose | 45.7 vs 52.4 vs 41.6 | Screening colonoscopy | Low residue | BBPS | 2 days before |
| Liu 2018 | Single center | Educational video vs SPI | 4L PEG | Split dose | 61.9 vs 62.5 | Mixed colonoscopy | Clear liquid | OBPS | 1 day before |
| Liu 2014 | Single center | Phone call vs SPI | 2L PEG, 1.5L sodium phosphate | Single dose | 33.7 vs 30.5 | Mixed colonoscopy | Clear liquid | OBPS | 1 day before |
| Lorenzo 2015 | Single center | Mobile app vs SPI | 2L PEG | Single dose | 28.7 vs 42.1 | Mixed colonoscopy | Low-fiber | HCS | n.r. |
| Meng 2015 | Single center | Additional explanation vs SPI | n.r. | n.r. | 0 | n.r. | n.r. | BBPS | 1 day before |
| Modi 2009 | Single center | Additional explanation vs SPI | 4L PEG with 3 bisacodyl | n.r. | 10.7 vs 21.3 | Screening colonoscopy | Clear liquid | UPAS | 21 days before |
| Park 2016 | Single center | Educational video vs SPI | 2L PEG | Split dose | 61.6 vs 56.7 | Screening colonoscopy | Clear liquid | OBPS | 1 day before |
| Pillai 2018 | Single center | Educational video vs SPI | Mixed | Mixed | n.r. | Screening colonoscopy | n.r. | OBPS | 30 days before |
| Prakash 2013 | Multicenter | Educational video vs SPI | 4L PEG | Split dose | 38.8 vs 56.1 | n.r. | n.r. | OBPS | 1 day before |
| Sharara 2017 | Single center | Mobile app vs SPI | Mixed | Split dose | 20.0 | Mixed colonoscopy | Clear liquid | ABPS | 3 days before |
| Spiegel 2011 | Single center | Newly designed booklet vs SPI | Mixed | mixed | 38.0 vs 36.8 | Mixed colonoscopy | n.r. | OBPS | 7 days before |
| Tae 2012 | Single center | New visual aids vs SPI | 4L PEG | Split dose | n.r. | Screening colonoscopy | Clear liquid | BBPS | n.r. |
| Walter 2019 | Multicenter | SMS vs SPI | 2L PEG plus Asc | Split dose | 35.1 vs 40.9 | Mixed colonoscopy | Low fiber | BBPS | 3 days before |
| Wang 2019 | Single center | SMS vs Social media app vs SPI | 3L PEG | Split dose | 37.2 vs 32.8 vs 40.2 | Mixed colonoscopy | Clear liquid | BBPS | 2 days before |
| Zhang 2018 | Single center | Social media app vs SPI | 3L PEG | n.r. | n.r. | Screening colonoscopy | n.r. | BBPS | 2 days before |
| Rice 2016 | Single center | Educational video vs SPI | 4L PEG | Mixed | 54.8 vs 48.0 | Mixed colonoscopy | Clear liquid | BBPS | 2-3 days before |

BP, bowel preparation; EPI, enhanced patient instruction; SPI, standard patient instructions; SMS, short message service; PEG, polyethylene glycol; SPMC, sodium picosulfate with magnesium citrate, BBPS, Boston Bowel Preparation Scle; ABPS, Aronchick Bowel Preparation Scale; OBPS, Ottawa Bowel Preparation Scale; HCS, Harefield Cleansing Scale; UPAS, Universal Preparation Assessment scale; n.r., not reported.
