# Supplementary material for: Comparative Effectiveness of Enhanced Patient Instructions for Bowel Preparation Before Colonoscopy: Network Meta-analysis of 23 Randomized Controlled Trials
Source: J Med Internet Res. 2021 Oct 25;23(10):e19915. doi: 10.2196/19915 (PMC8576559; doi:10.2196/19915)
Supplement: Multimedia Appendix 4 [file jmir_v23i10e19915_app4.docx]

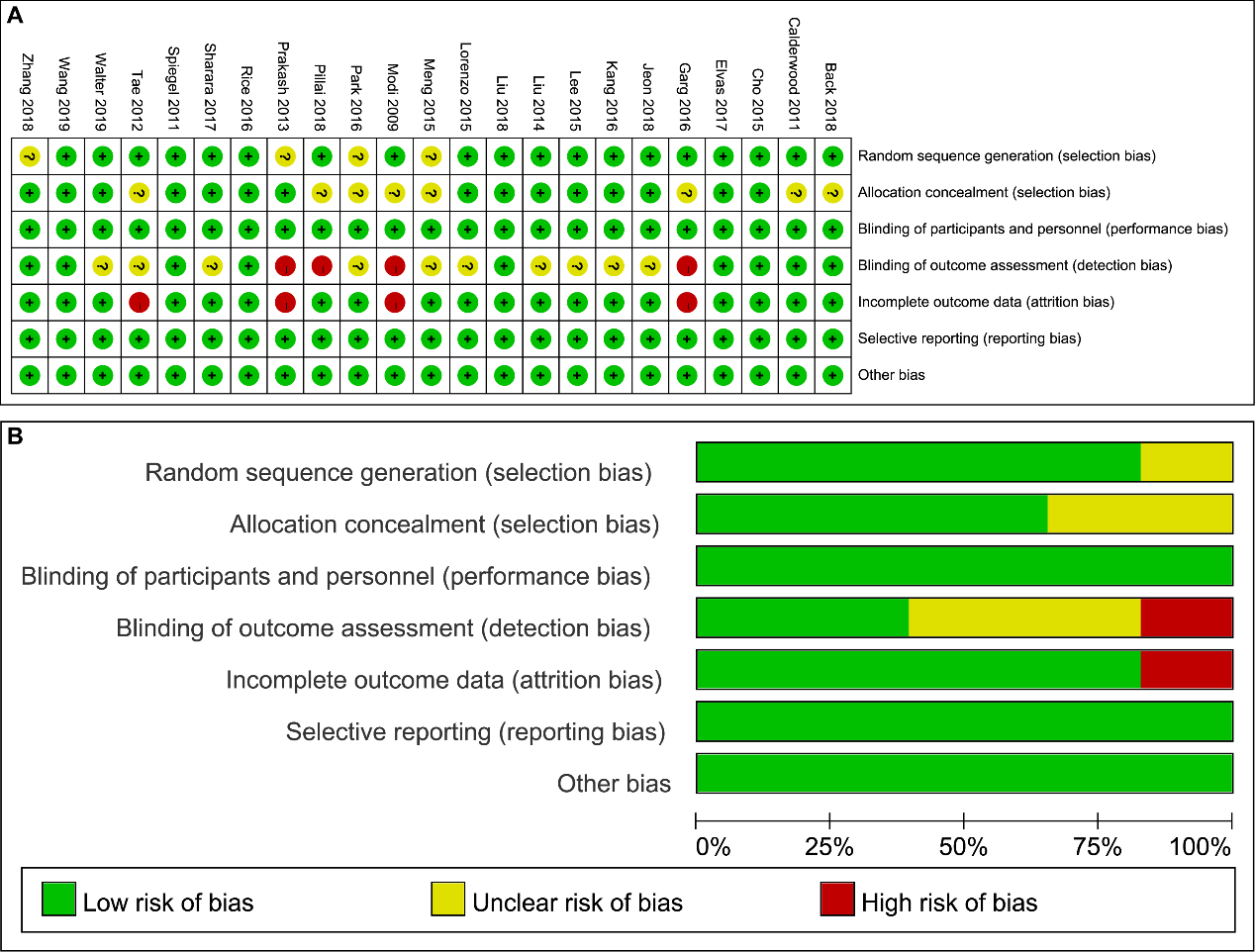


**Figure S1.** Risk of bias of study-level and overall quality assessments. Green, yellow or red represents low, unclear or high risk of bias.


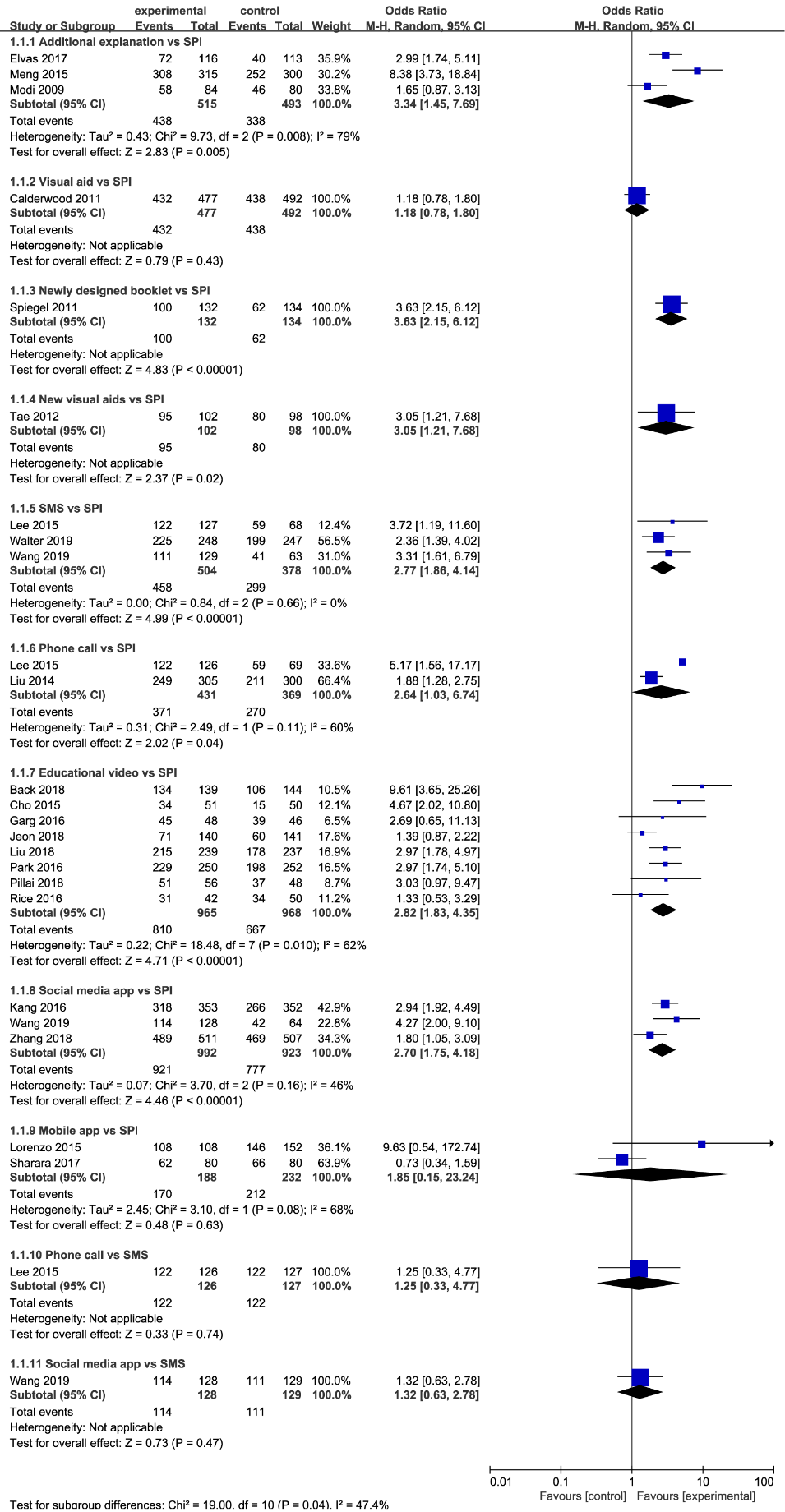


**Figure S2.** Meta-analysis of APR. The summary effect estimates odds ratio (OR) for individual randomized controlled trial (RCT) are indicated by blue rectangles (the size of the rectangle is proportional to the study weight), with the black horizontal lines representing 95% confidence interval (CI). The overall summary effect estimate (OR) and 95% CI are indicated by the black diamond below. SMS, short message service; SPI, standard patient instruction; APR, adequate preparation rate; M-H, Mantel-Haenszel.


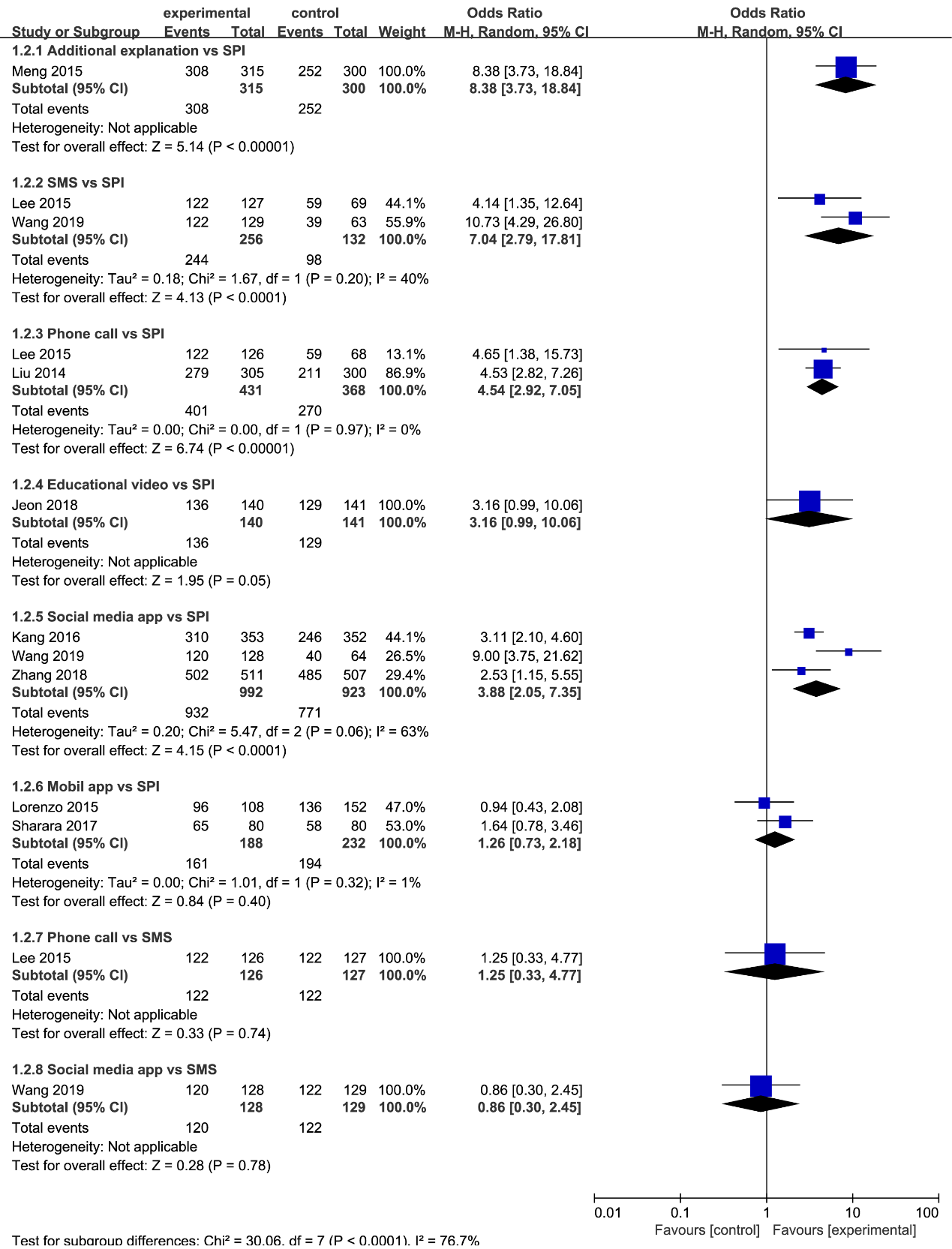


**Figure S3.** Meta-analysis of AI. The summary effect estimates odds ratio (OR) for individual randomized controlled trial (RCT) are indicated by blue rectangles (the size of the rectangle is proportional to the study weight), with the black horizontal lines representing 95% confidence interval (CI). The overall summary effect estimate (OR) and 95% CI are indicated by the black diamond below. SMS, short message service; SPI, standard patient instruction; AI, adherence to instruction; M-H, Mantel-Haenszel.


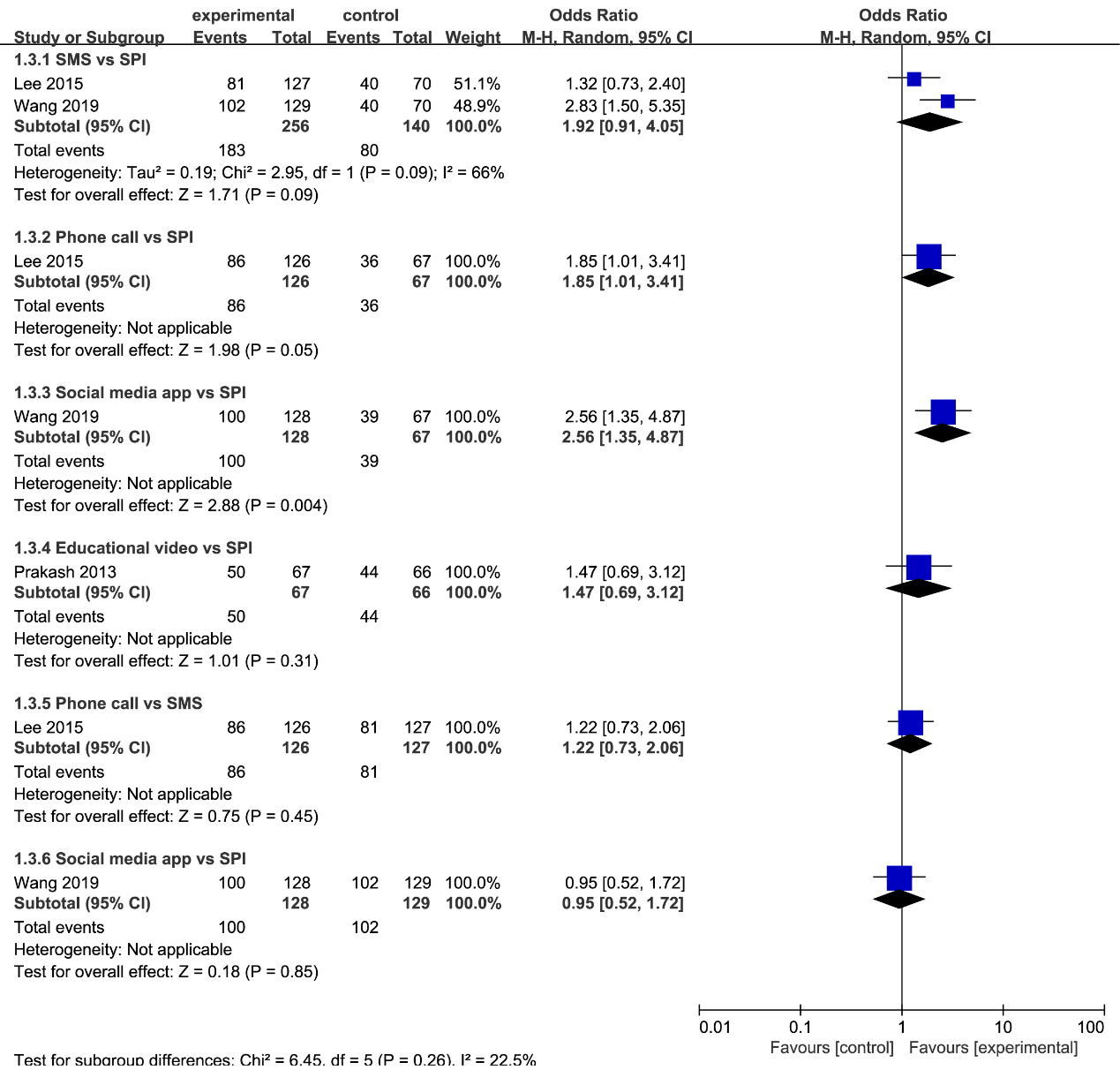


**Figure S4.** Meta-analysis of SWBP. The summary effect estimates odds ratio (OR) for individual randomized controlled trial (RCT) are indicated by blue rectangles (the size of the rectangle is proportional to the study weight), with the black horizontal lines representing 95% confidence interval (CI). The overall summary effect estimate (OR) and 95% CI are indicated by the black diamond below. SMS, short message service; SPI, standard patient instruction; SWBP, satisfaction with bowel preparation solution; M-H, Mantel-Haenszel.


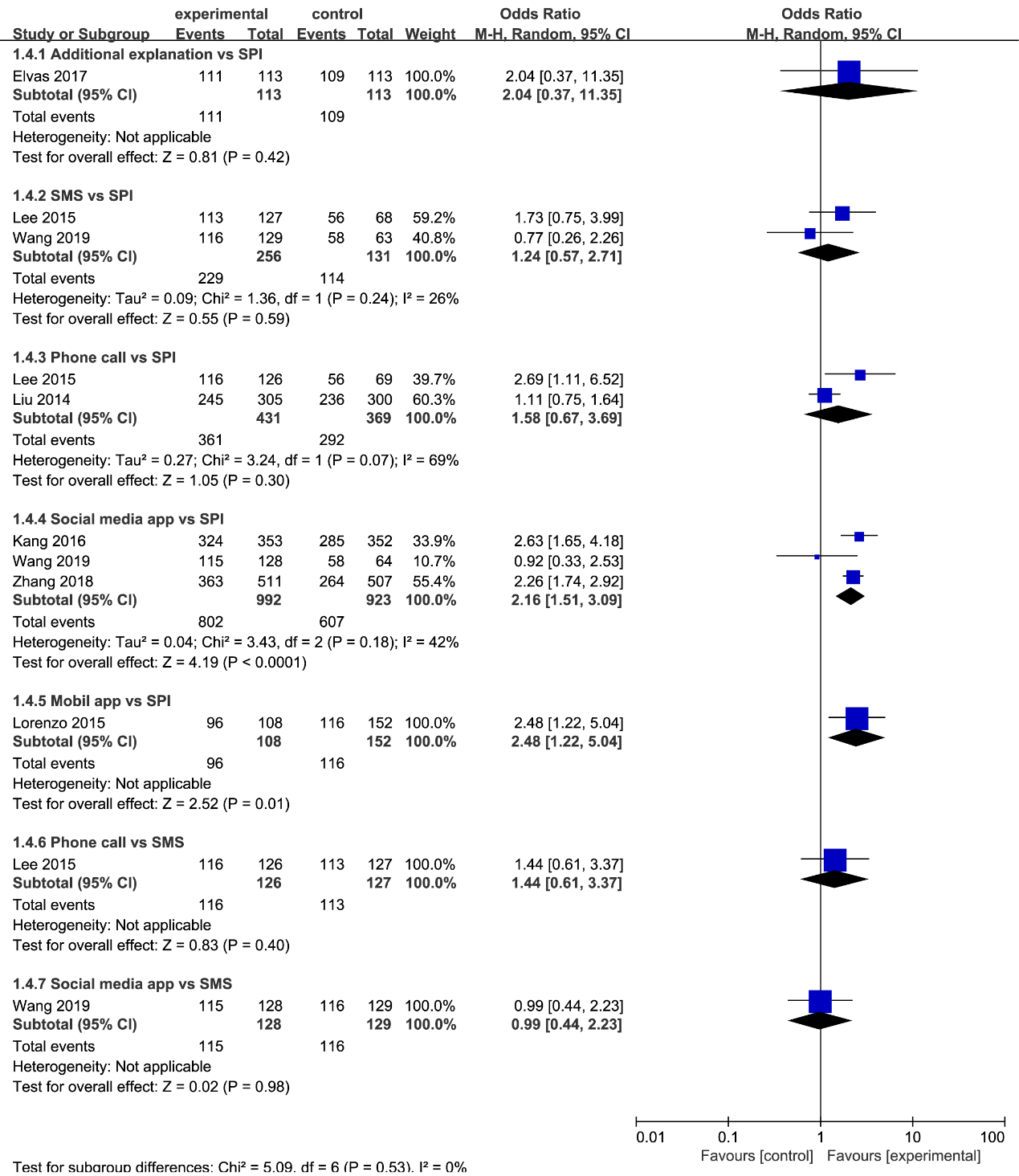


**Figure S5.** Meta-analysis of WRBP. The summary effect estimates odds ratio (OR) for individual randomized controlled trial (RCT) are indicated by blue rectangles (the size of the rectangle is proportional to the study weight), with the black horizontal lines representing 95% confidence interval (CI). The overall summary effect estimate (OR) and 95% CI are indicated by the black diamond below. SMS, short message service; SPI, standard patient instruction; WRBP, willingness to repeat the same preparation solution; M-H, Mantel-Haenszel.


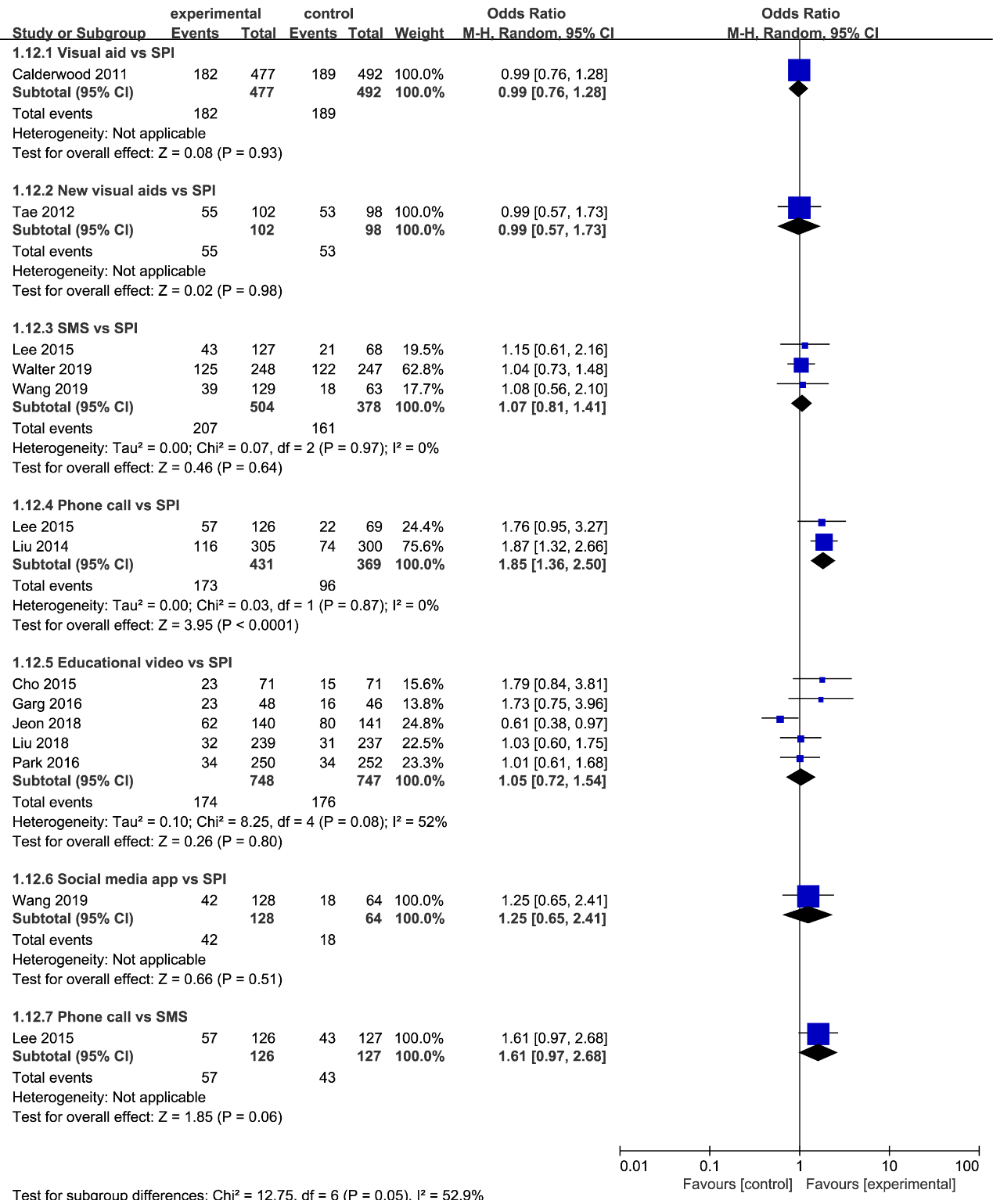


**Figure S6.** Meta-analysis of PDR. The summary effect estimates odds ratio (OR) for individual randomized controlled trial (RCT) are indicated by blue rectangles (the size of the rectangle is proportional to the study weight), with the black horizontal lines representing 95% confidence interval (CI). The overall summary effect estimate (OR) and 95% CI are indicated by the black diamond below. SMS, short message service; SPI, standard patient instruction; PDR, polyp detection rate; M-H, Mantel-Haenszel.


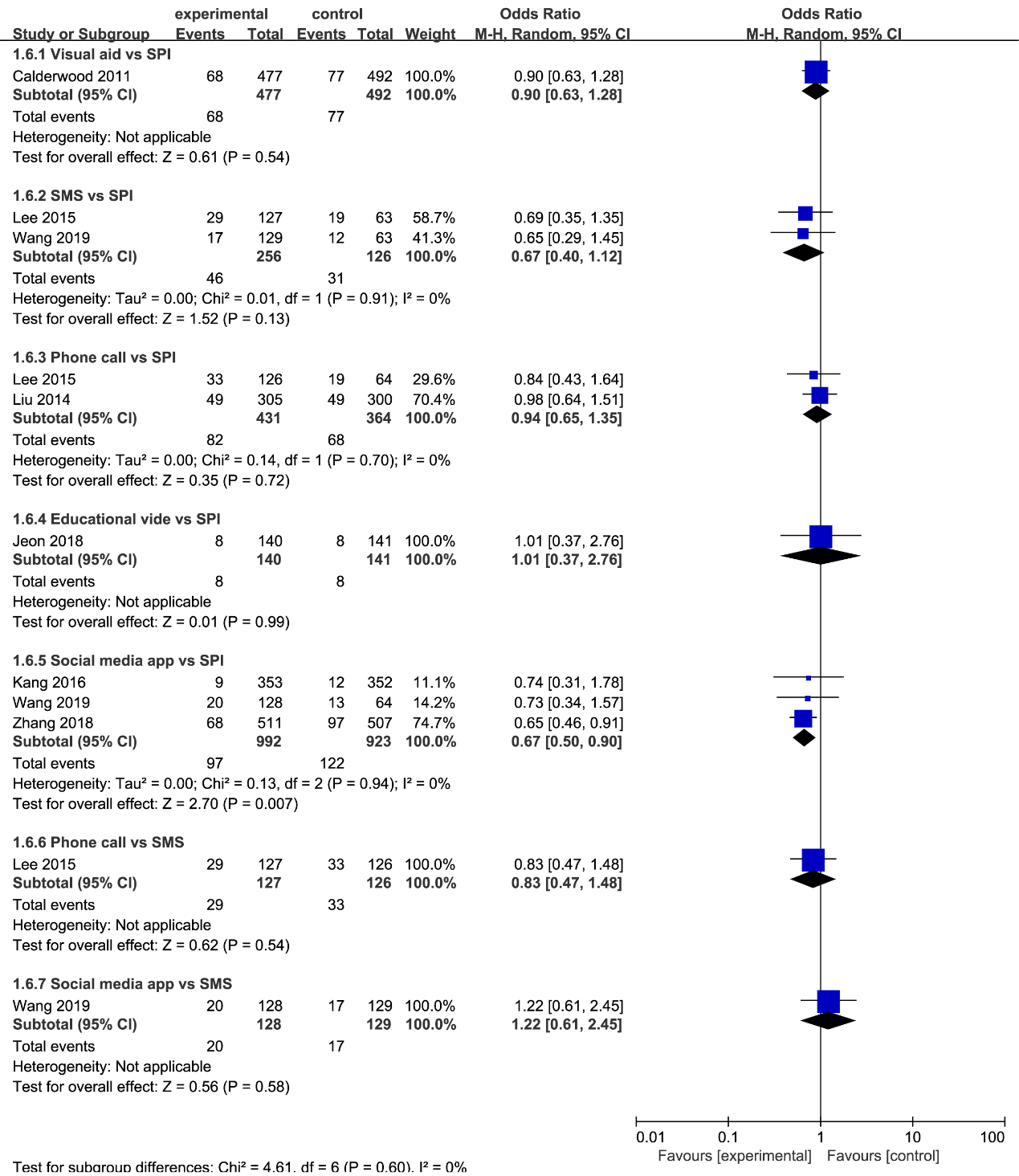


**Figure S7.** Meta-analysis of AD. The summary effect estimates odds ratio (OR) for individual randomized controlled trial (RCT) are indicated by blue rectangles (the size of the rectangle is proportional to the study weight), with the black horizontal lines representing 95% confidence interval (CI). The overall summary effect estimate (OR) and 95% CI are indicated by the black diamond below. SMS, short message service; SPI, standard patient instruction; AD, abdominal discomfort; M-H, Mantel-Haenszel.


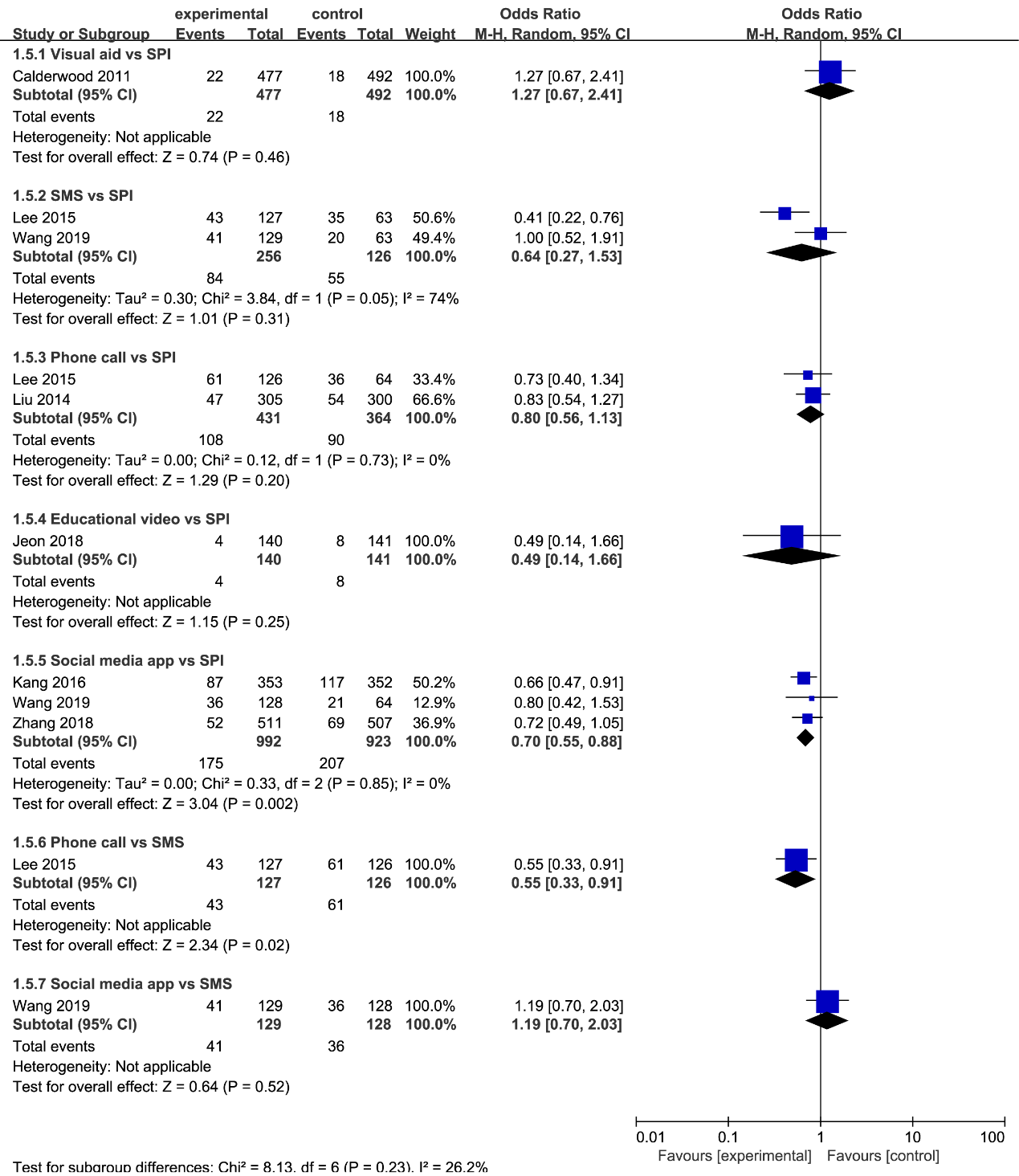


**Figure S8.** Meta-analysis of NV. The summary effect estimates odds ratio (OR) for individual randomized controlled trial (RCT) are indicated by blue rectangles (the size of the rectangle is proportional to the study weight), with the black horizontal lines representing 95% confidence interval (CI). The overall summary effect estimate (OR) and 95% CI are indicated by the black diamond below. SMS, short message service; SPI, standard patient instruction; NV, nausea and vomiting; M-H, Mantel-Haenszel.


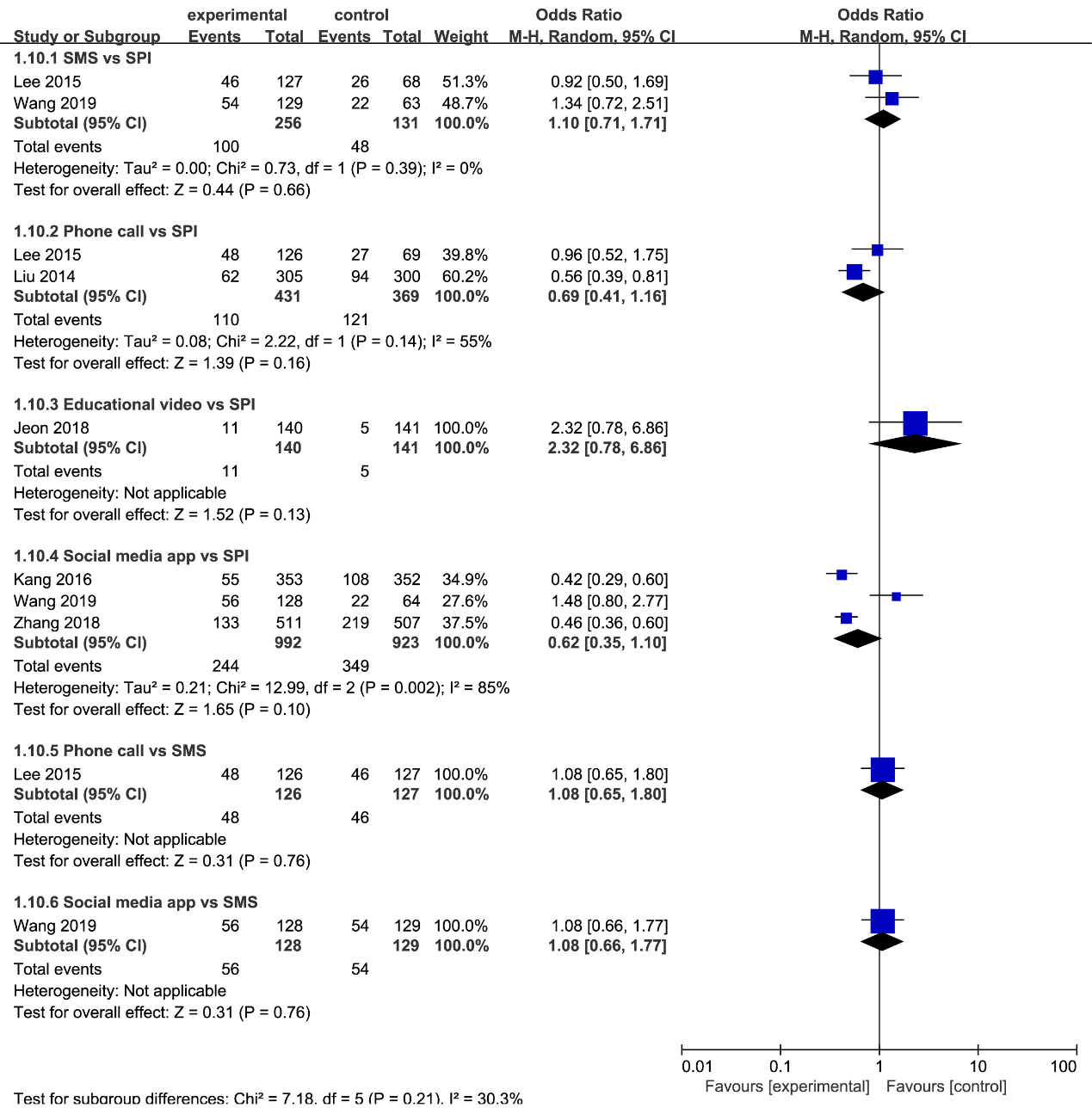


**Figure S9.** Meta-analysis of SDT. The summary effect estimates odds ratio (OR) for individual randomized controlled trial (RCT) are indicated by blue rectangles (the size of the rectangle is proportional to the study weight), with the black horizontal lines representing 95% confidence interval (CI). The overall summary effect estimate (OR) and 95% CI are indicated by the black diamond below. SMS, short message service; SPI, standard patient instruction; SDT, sleep disturbance; M-H, Mantel-Haenszel.


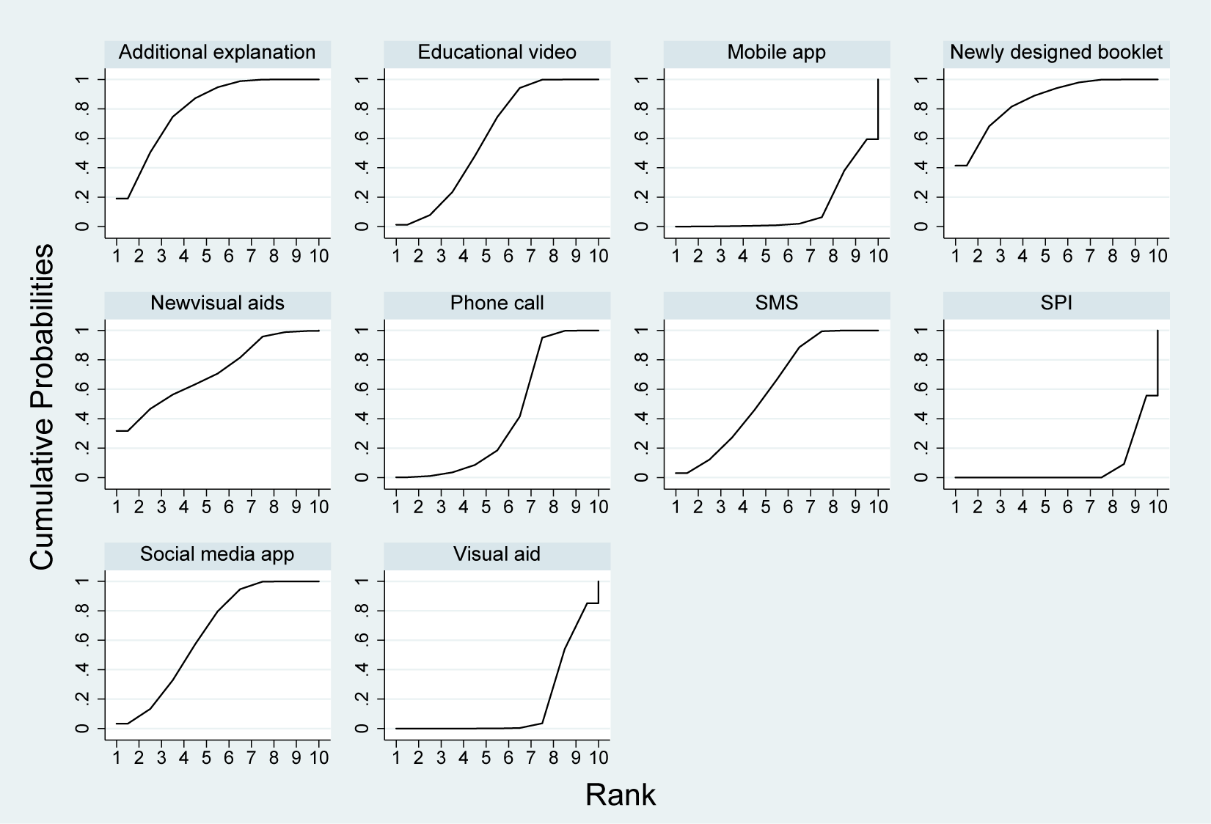


**Figure S10.** Rank of all patient instructions in terms of APR according to cumulative probability. APR, adequate preparation rate.
