# Supplementary material for: Comparative Effectiveness of Enhanced Patient Instructions for Bowel Preparation Before Colonoscopy: Network Meta-analysis of 23 Randomized Controlled Trials
Source: J Med Internet Res. 2021 Oct 25;23(10):e19915. doi: 10.2196/19915 (PMC8576559; doi:10.2196/19915)
Supplement: Multimedia Appendix 5 [file jmir_v23i10e19915_app5.docx]

| **Supplementary Table 4.** Sensitivity analysis | | | | | | | |
| --- | --- | --- | --- | --- | --- | --- | --- |
| Education instruction | Adequatepreparation rate | | | Adherence to instruction | Willingness to repeat | Ployp detection rate | |
|  | Scale^a^ | High risk^b^ | Design^c^ | Scale^a^ | Scale^a^ | High risk^b^ | Design^c^ |
| Compared with SPI |  |  |  |  |  |  |  |
| Additional explanation | **4.48 (2.87-6.70)** | **4.49 (2.88-6.80)** | **3.56 (2.45-5.03)** | **9.80 (4.06-21.92)** | 1.04 (0.18-3.27) | - | - |
| Visual aid | 1.21 (0.78-1.81) | 1.22 (0.78-1.81) | 1.22 (0.78-1.81) | - | - | 1.00 (0.76-1.28) | 1.00 (0.76-1.29) |
| Newly designed booklet | **3.81 (2.16-6.27)** | **3.78 (2.19-6.24)** | **3.80 (2.18-6.26)** | - | - | - | - |
| New visual aids | **3.66 (1.31-8.79)** | - | **3.58 (1.29-8.45)** | - | - | - | 1.04 (0.57-1.73) |
| SMS | **2.70 (1.85-3.85)** | **2.71 (1.85-3.89)** | **3.06 (1.79-5.03)** | **7.04 (3.55-13.12)** | 1.44 (0.86-2.30) | 1.09 (0.84-1.39) | 1.17 (0.81-1.62) |
| Phone call | **2.15 (1.50-3.03)** | **2.16 (1.50-3.04)** | **2.17 (1.49-3.06)** | **4.85 (3.11-7.35)** | 1.36 (0.94-1.89) | **1.86 (1.39-2.42)** | **1.88 (1.41-2.47)** |
| Educational video | **2.70 (2.12-3.41)** | **2.69 (2.10-3.43)** | **2.71 (2.12-3.43)** | **4.25 (1.10-12.65)** | - | 0.81 (0.60-1.06) | 0.81 (0.60-1.07) |
| Social media app | **2.81 (2.07-3.76)** | **2.80 (2.07-3.72)** | **2.83 (2.07-3.79)** | **3.76 (2.70-5.11)** | **2.22 (1.78-2.74)** | 1.26 (0.76-1.98) | 1.40 (0.89-2.09) |
| Mobile app | 0.79 (0.33-1.59) | 1.13 (0.52-2.17) | 1.13 (0.53-2.18) | 1.80 (0.79-3.60) | - | - | - |
| Compared with additional explanation |  |  |  |  |  |  |  |
| Visual aid | **0.28 (0.15-0.49)** | **0.28 (0.15-0.49)** | **0.35 (0.19-0.59)** | - | - | - | - |
| Newly designed booklet | 0.89 (0.43-1.66) | 0.89 (0.42-1.64) | 1.10 (0.56-2.00) | - | - | - | - |
| New visual aids | 0.86 (0.27-2.23) | - | 1.04 (0.34-2.58) | - | - | - | - |
| SMS | 0.63 (0.35-1.07) | 0.63 (0.34-1.07) | 0.89 (0.45-1.61) | 0.86 (0.25-2.12) | 2.37 (0.39-8.07) | - | - |
| Phone call | **0.50 (0.28-0.84)** | **0.50 (0.28-0.84)** | 0.63 (0.37-1.01) | 0.59 (0.20-1.32) | 2.23 (0.39-7.45) | - | - |
| Educational video | **0.63 (0.38-0.99)** | **0.63 (0.37-0.99)** | 0.79 (0.50-1.19) | 0.52 (0.09-1.79) | - | - | - |
| Social media app | 0.66 (0.38-1.07) | 0.66 (0.37-1.06) | 0.82 (0.50-1.27) | **0.46 (0.16-0.99)** | 3.65 (0.66-12.02) | - | - |
| Mobile app | **0.19 (0.07-0.41)** | **0.26 (0.11-0.56)** | **0.33 (0.14-0.67)** | **0.22 (0.06-0.56)** | - | - | - |
| Compared with visual aid |  |  |  |  |  |  |  |
| Newly designed booklet | **3.29 (1.58-6.11)** | **3.26 (1.57-6.03)** | **3.27 (1.58-6.12)** | - | - | - | - |
| New visual aids | **3.16 (1.02-8.01)** | - | 3.09 (0.97-7.70) | - | - | - | 1.06 (0.54-1.86) |
| SMS | **2.34 (1.28-3.91)** | **2.33 (1.28-3.91)** | **2.63 (1.29-4.87)** | - | - | 1.11 (0.76-1.57) | 1.19 (0.75-1.79) |
| Phone call | **1.86 (1.02-3.10)** | **1.85 (1.03-3.06)** | **1.87 (1.03-3.11)** | - | - | **1.89 (1.27-2.73)** | **1.92 (1.28-2.76)** |
| Educational video | **2.34 (1.40-3.67)** | **2.32 (1.39-3.67)** | **2.34 (1.41-3.68)** | - | - | 0.82 (0.55-1.20) | 0.82 (0.55-1.20) |
| Social media app | **2.42 (1.39-3.90)** | **2.41 (1.41-3.87)** | **2.44 (1.41-3.95)** | - | - | 1.29 (0.72-2.14) | 1.42 (0.83-2.26) |
| Mobile app | 0.68 (0.25-1.48) | 0.97 (0.39-2.03) | 0.97 (0.39-2.04) | - | - | - | - |
| Compared with newly designed booklet |  |  |  |  |  |  |  |
| New visual aids | 1.03 (0.31-2.71) | - | 1.02 (0.30-2.62) | - | - | - | - |
| SMS | 0.76 (0.38-1.39) | 0.77 (0.38-1.38) | 0.86 (0.39-1.68) | - | - | - | - |
| Phone call | 0.61 (0.30-1.09) | 0.61 (0.31-1.09) | 0.61 (0.31-1.09) | - | - | - | - |
| Educational video | 0.76 (0.41-1.30) | 0.76 (0.41-1.29) | 0.77 (0.41-1.31) | - | - | - | - |
| Social media app | 0.79 (0.41-1.39) | 0.80 (0.42-1.37) | 0.80 (0.42-1.39) | - | - | - | - |
| Mobile app | **0.22 (0.08-0.51)** | **0.32 (0.12-0.70)** | **0.32 (0.12-0.70)** | - | - | - | - |
| Compared with new visual aids |  |  |  |  |  |  |  |
| SMS | 0.93 (0.29-2.20) | - | 1.07 (0.31-2.64) | - | - | - | 1.22 (0.60-2.22) |
| Phone call | 0.75 (0.23-1.74) | - | 0.76 (0.24-1.78) | - | - | - | 1.97 (1.00-3.49) |
| Educational video | 0.93 (0.29-2.12) | - | 0.95 (0.31-2.16) | - | - | - | 0.85 (0.43-1.52) |
| Social media app | 0.97 (0.30-2.24) | - | 1.00 (0.32-2.30) | - | - | - | 1.46 (0.68-2.76) |
| Mobile app | **0.27 (0.06-0.75)** | - | 0.40 (0.10-1.05) | - | - | - | - |
| Compared with SMS |  |  |  |  |  |  |  |
| Phone call | 0.82 (0.49-1.31) | 0.82 (0.48-1.32) | 0.76 (0.39-1.31) | 0.77 (0.32-1.52) | 1.00 (0.55-1.65) | **1.73 (1.21-2.40)** | **1.66 (1.10-2.41)** |
| Educational video | 1.04 (0.65-1.56) | 1.03 (0.64-1.56) | 0.95 (0.51-1.58) | 0.67 (0.14-2.19) | - | 0.76 (0.51-1.08) | 0.72 (0.70-1.10) |
| Social media app | 1.07 (0.67-1.62) | 1.07 (0.66-1.62) | 0.99 (0.54-1.63) | 0.59 (0.27-1.09) | 1.65 (0.93-2.67) | 1.17 (0.70-1.83) | 1.22 (0.76-1.87) |
| Mobile app | **0.30 (0.11-0.65)** | **0.43 (0.18-0.90)** | **0.40 (0.15-0.86)** | **0.29 (0.09-0.69)** | - | - | - |
| Compared with phone call |  |  |  |  |  |  |  |
| Educational video | 1.30 (0.83-1.93) | 1.29 (0.82-1.94) | 1.29 (0.82-1.94) | 0.92 (0.21-2.87) | - | **0.44 (0.29-0.65)** | **0.44 (0.29-0.64)** |
| Social media app | 1.35 (0.82-2.07) | 1.34 (0.83-2.07) | 1.35 (0.82-2.08) | 0.81 (0.46-1.33) | **1.69 (1.11-2.48)** | 0.69 (0.39-1.14) | 0.76 (0.45-1.20) |
| Mobile app | 0.38 (0.14-.81) | 0.54 (0.22-1.12) | 0.54 (0.23-1.11) | **0.39 (0.15-0.85)** | - | - | - |
| Compared with educational video |  |  |  |  |  |  |  |
| Social media app | 1.05 (0.71-1.51) | 1.06 (0.70-1.52) | 1.06 (0.71-1.52) | 1.31 (0.28-3.59) | - | 1.59 (0.88-2.66) | **1.76 (1.02-2.87)** |
| Mobile app | **0.30 (0.12-0.61)** | **0.43 (0.19-0.84)** | **0.42 (0.19-0.83)** | 0.63 (0.11-1.93) | - | - | - |
| Compared with social media app |  |  |  |  |  |  |  |
| Mobile app | **0.29 (0.11-0.61)** | **0.41 (0.18-0.83)** | **0.41 (0.18-0.83)** | 0.49 (0.20-1.03) | - | - | - |

Sensitivity analysis was based on (a) bowel assessment scale (excluding studies in which uncommon scale was used); (b) risk of bias (excluding studies with high risk); and (c) study design (excluding studies with multicenter design). The analysis is based on combined direct and indirect evidence from Bayesian network meta-analysis with different educational instructions in patients undergoing colonoscopy. Numbers in parentheses represent the 95% credible intervals (Crl). The column instruction is compared with the row instruction (ie, row instruction is reference for each comparison). Numbers in parentheses indicate 95% Crl.
